# Spatial Transcriptomics and Single Cell‐RNASeq Reveals Cellular Heterogeneity of SARS‐CoV‐2 in Lung Tissues and Global Mutational Patterns in COVID‐19 Patients

**DOI:** 10.1002/jmv.70586

**Published:** 2025-09-05

**Authors:** Seyed Taleb Hosseini, Mohammadamin Mahmanzar, Karim Rahimian, Saleha Bayat, Amir Gholamzad, Mahsa Mollapour Sisakht, Amin Farhadi, Donna Lee Kuehu, Youping Deng

**Affiliations:** ^1^ Medicinal Plants Research Center, Institute of Herbal Medicines and Metabolic Disorders Mazandaran University of Medical Sciences Sari Iran; ^2^ Department of Quantitative Health Sciences, John A. Burns School of Medicine University of Hawaii at Manoa Honolulu Hawaii USA; ^3^ Institute of Biochemistry and Biophysics (IBB) University of Tehran Tehran Iran; ^4^ Department of Biology, Research Center for Animal Development Applied Biology, Mashhad Branch Islamic Azad University Mashhad Iran; ^5^ Farhikhtegan Medical Convergence Sciences Research Center, Farhikhtegan Hospital,Te.Ms.C Islamic Azad University Tehran Iran; ^6^ Faculty of Pharmacy, Biotechnology Research Center Tehran University of Medical Sciences Tehran Iran; ^7^ Department of Biology Payame Noor University Tehran Iran

**Keywords:** amino acid, epidemiology, mutation, SARS‐CoV2, single cell RNASeq, spatial transcriptomics

## Abstract

RNA viruses have high mutation frequency, quick generation periods and vast population numbers, which promote fast evolution and host environment adaptation. We integrated scRNA‐seq and spatial transcriptomics to profile immune cells and viral gene expression in COVID‐19. Cell types and interactions were identified using Seurat‐based tools. Spatial transcriptomics analysis revealed viral hotspots, and GISAID data were used to track SARS‐CoV‐2 mutations. Single‐cell and spatial transcriptomics analyses revealed that immune cells such as Neutrophils, Monocyte:CD14 + , and T cell:CD4+ central memory are highly abundant in COVID‐19 patients, particularly in mild and severe cases, and are concentrated in the central and upper regions of lung tissue. Pseudotime and CellChat analyses indicated that cell differentiation trajectories and communication networks shift toward heightened inflammatory responses in severe conditions. Spatial analysis of viral gene expression showed that SARS‐CoV‐2 genes, especially Nucleoprotein, Spike and Envelope were highly expressed in central and upper‐right tissue regions, suggesting active viral replication. This localized viral activity was strongly associated with areas of immune cell infiltration and inflammation. The top 10 sustainable mutants in SARS‐CoV‐2 genome with high frequency were observed in NSP12 (P323L, 99%, Switzerland), Spike (D614G, 97%, Switzerland), NSP4 (T492I, 79%, Switzerland), NSP6 (T77A, 70%, Guangdong), Orf9c (G50N, 64%, England), Nucleoprotein (D377Y, 62%, United States), Orf9b (T60A, 61%, France), NSP14 (I42V, 55%, United States), Envelope (T9I, 51.3%, Trinidad and Tobago), and NSP5 (P132H, 51.2%, United States). Following to this approach is crucial for a strong epidemiological reaction against the changing SARS‐CoV‐2 outbreak.

## 1 Introduction

In the SARS‐CoV‐2 virus, phenotypic changes and genetic variants are strongly associated with virus behaviors [1]. The genome of SARS‐CoV‐2 is a single‐stranded positive‐sense RNA that encodes nonstructural proteins (NSPs) and structural proteins [2]. The structural proteins encompass the Spike (S), Envelope (E) and Membrane (M), as well as the Nucleocapsid (N) protein that bundles the viral genome. NSP1 is a major determinant in viral pathogenicity and additionally, since NSP1 plays a critical function in the coronavirus disease 2019 (COVID‐19), monitoring any structural alteration is crucial [3]. There are six main readout frames in the virus genome open reading frame (Orf). The 3′termini of the genome are composed of four structural proteins: S glycoprotein, E protein, M glycoprotein and N phosphoprotein [4]. The angiotensin‐converting enzyme 2 (ACE2) is the primary entry receptor for the S protein in SARS‐CoV‐2, facilitating the virus entry [5, 6, 7]. Studies indicate that the COVID‐19 pandemic's main agent, the SARS‐CoV‐2 lineage, acquires one to two mutations every month as it spreads through subsequent hosts, promoting infection, pathogenic, or immunity avoidance [8, 9]. In most cases, these mutations do not affect pathogenicity and could only affect dissemination [5, 6, 10]. N501Y, E484K, and D614G are examples of common mutations between variants [5, 6, 10, 11]. SARS‐CoV‐2 has over 10,000 distinct mutations compared to the reference genome [12]. From the 12,509 viral genomes of SARS‐CoV‐2 sequences analyzed thus far in the COVID‐19 pandemic outbreak, the CoV‐GLUE project (http://cov-glue.cvr.gla.ac.uk/#/home) has identified 5,033 amino acid replacements, with 1,687 mutations found in the S glycoprotein, 334 in the N phosphoprotein, 95 in the M glycoprotein and 45 in the E protein [4]. So far, stable mutants have been identified for Alpha (B.1.1.7 and Q lineages), Beta (B.1.351 and descendent lineages), Gamma (P.1 and descendent lineages), Epsilon (B.1.427 and B.1.429), Eta (B.1.525), Iota (B.1.526), Kappa (B.1.617.1, 1.617.3), Mu (B.1.621, B.1.621.1), and Zeta (B.1.1.529 and BA lineages) (https://www.cdc.gov/coronavirus/2019-ncov/variants/variant-classifications.html) [13, 14]. Furthermore, geographically distinct SARS‐CoV‐2 etiological effects can be attributed to the genetic heterogeneity of SARS‐CoV‐2 specimens worldwide [15]. There are potentially several SARS‐CoV‐2 different types with various infection frequency capabilities, which are demonstrated by the large number of distinct S protein mutations [12]. For instance, the Delta variant reduces the efficacy of therapies more than the other variants [16]. Furthermore, viral mutation studies can help develop new vaccines, antiviral drugs and diagnostic assays. Although several databases have investigated gene‐specific mutations, including CoV‐Spectrum [17], CoVariants (https://covariants.org/), CoVerage [18], CoVizu [19], Covid‐Miner (https://rupertoverall.net/covidminer/) and CNCB (https://www.cncb.ac.cn/), they have not examined the geographical regions that are most likely to be susceptible to long‐term mutations. Novel approaches including single‐cell and spatial transcriptomics facilitate high‐resolution cellular gene expression studies, which provides insights into the origin of disease [20]. Single‐cell RNA sequencing in viral infections such as SARS‐CoV‐2 identifies important types of cells associated with viral spread and inflammatory processes and demonstrates variation in host immune responses [20]. This is complemented by spatial transcriptomics, which maps gene expression within the structure of tissues to identify immunological communications and targeted viral impacts in areas of infection [20]. These approaches have been crucial in determining therapeutic targets, comprehending the evolution of COVID‐19 and establishing the mechanisms underlying tissue damage and viral transmission.

To comprehensively investigate the cellular, spatial, and genomic dynamics of SARS‐CoV‐2 infection, we employed an integrative approach combining single‐cell RNA seq (scRNA‐seq), spatial transcriptomics, and large‐scale viral mutation analysis. The study was structured around three central aims: (1) To identify immune correlates of COVID‐19 severity, we performed scRNA‐seq on peripheral blood samples from three groups: Healthy (*n* = 4; 21 294 genes; 23 389 cells), Mild (*n* = 4; 21 376 genes; 17 025 cells), and Severe (*n* = 4; 20 349 genes; 14 229 cells). Comparative analyses between healthy versus mild and healthy versus severe groups were conducted to characterize immune cell heterogeneity and disease‐associated transcriptional profiles. (2) To map the tissue‐level immune landscape and viral burden, we integrated scRNA‐seq findings with spatial transcriptomics data from five lung tissue samples, including three COVID‐19‐infected lungs (V10B13‐400, V10B13‐401, V10L13‐003) and two healthy controls (V10S29‐079, V10S29‐080). This enabled spatial localization of immune cell types and detailed analysis of viral gene expression (e.g., ORF1ab, N, ORF10) across different lung regions. (3) To examine global mutational trends in SARS‐CoV‐2, we analyzed 15 669 529 viral genome samples from different countries and continents and tracking the emergence and prevalence of mutations over time. Collectively, our study aims to link immune responses at the single‐cell and tissue level with patterns of viral gene expression and genomic evolution, offering a multi‐dimensional understanding of COVID‐19 pathogenesis and informing future therapeutic and diagnostic strategies.

## 2 Methods

### 2.1 Detection of Single Cell and Spatial Transcriptomics Datasets

**Figure 1:**
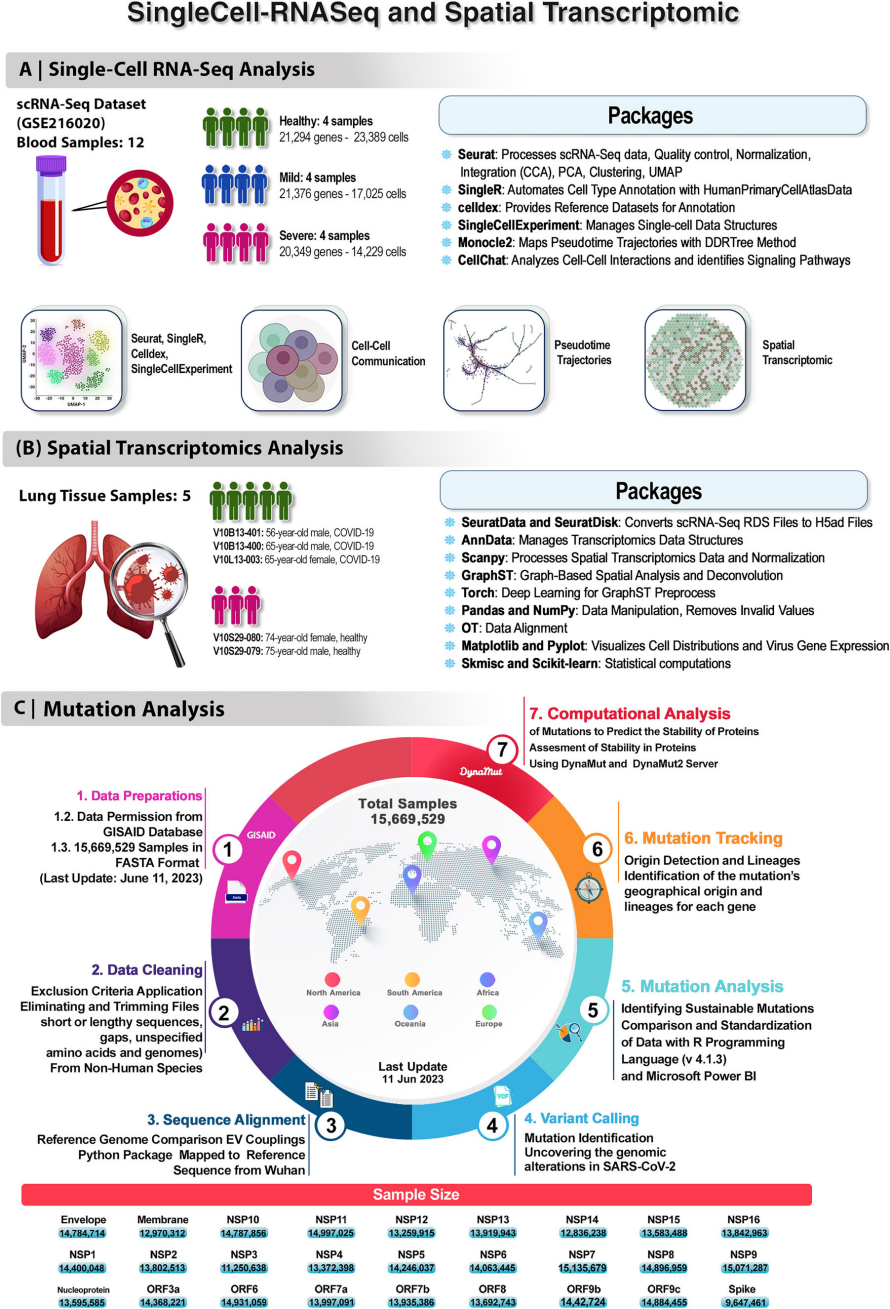
Omics approaches in this study. (A). Single cell RNASeq analysis of 12 blood samples including: four healthy (21 294 genes and 23 389 cells), four mild (21 376 genes and 17 025 cells) and four severe (20 349 genes and 14 229 cells) from patients with covid‐19. (B). Spatial transcriptomics analysis of 5 lung tissue samples from patients with covid‐19 such as: three lung tissue samples (covid‐19: V10B13‐401, V10B13‐400, V10L13‐003) and two lung tissue samples (control: V10S29‐080, V10S29‐079). (C). Mutation detection approaches and number of samples for all proteins related to the covid‐19.

The general workflow of data analyzing and detailed approaches are explained in Figure 1. We investigated the PubMed database, the Gene Expression Omnibus database (GEO, https://www.ncbi.nlm.nih.gov/geo/) [21] and the Sequence Read Archive (SRA, https://www.ncbi.nlm.nih.gov/sra) [22] to find single cell RNA‐Seq and spatial transcriptomics depending covid‐19 gene expression profiling studies involving lung and blood samples. The search was conducted using the following keywords: “gene expression, SARS‐CoV‐2, COVID‐19, single cell analysis, spatial transcriptomics, Visium gene expression, immune cell profiling, viral gene expression and viral mutations”. We included single‐cell RNA‐Seq datasets related to gene expression profiles of immune and lung tissue cell types from COVID‐19 patients, as well as healthy controls. Excluded were single‐cell RNA‐Seq datasets from studies involving animal models such as mice and ferrets, studies involving antiviral treatments or antibody‐based interventions on cell lines and systematic review publications.

### 2.2 Number of Datasets and Samples for Single Cell and Spatial Transcriptomic

The GSE216020 [23] data set consists of 12 single‐cell samples carefully organized according to the severity of the disease (Mild and Severe). It includes four normal samples that together contain 21 294 genes and 23 389 cells, as well as four mild samples with 21 376 genes and 17 025 cells, and four severe samples encompassing 20 349 genes and 14 229 cells. Complementing this single‐cell data is a spatial data set accessible for download through the link https://doi.org/10.5281/zenodo.8039011 [24]. This spatial data set comprises five distinct samples. The first sample, known as V10B13‐401, was obtained from a 56‐year‐old man diagnosed with COVID‐19. The second sample, referred to as V10B13‐400, was sourced from a 65‐year‐old man also affected by COVID‐19. The third sample, designated V10L13‐003, comes from a 65‐year‐old woman who was diagnosed with COVID‐19. The fourth sample, identified as V10S29‐080, was collected from a healthy 74‐year‐old woman. Finally, the fifth sample, labeled V10S29‐079, was derived from a healthy 75‐year‐old man.

### 2.3 Single‐Cell Assessment for Quality Control (QC) and Data Integration

The 10xGenomice scRNA‐seq data was analyzed using the Seurat package [25] in the R programming language. After applying QC to the raw matrix and removing low‐quality cells according to the following criteria, a high‐quality scRNA‐seq expression matrix was generated: (1) Specifically, cells expressing more than 200 different genes and genes expressed in at least three different cells were determined to be appropriate for utilization during the construction of a Seurat object. (2) Only cells with gene expression levels of greater than 200 and less than 5000 for healthy objects, 4500 for mild objects, and 5000 for severe objects have been included overall. (3) To find the number of genes related to either ribosomal or mitochondrial activity which have been found in each cell, the “PercentageFeatureSet” function was employed. The “LogNormalize” method in the “NormalizeData” function was used to normalize the scRNA‐seq data. The top 2000 highly variable genes were chosen and displayed using the “FindVariableFeatures” and “VariableFeaturePlot” functions after quality control. The “SelectIntegrationFeatures,” “FindIntegrationAnchors” and “IntegrateData” methods were used to integrate all Seurat objects (healthy, mild, and severe) using the CCA algorithm. Batch effect correction was performed using the Seurat integration workflow. Specifically, “SelectIntegrationFeatures” function was used to identify shared variable genes, “FindIntegrationAnchors” function was utilized to detect anchors across samples, and “IntegrateData” function was applied to integrate and correct for batch effects. These steps ensured the alignment of shared biological signals across different scRNA‐seq samples before downstream analysis. The data was then scaled and centered for each gene expression using the “ScaleData” function. Consequently, a variance of 1 and a mean expression of 0 were obtained inside the cell.

### 2.4 Nonlinear Dimension Reduction and Unsupervised Cluster Analysis

Principal component analysis (PCA) was used to examine 2000 genes employing the “RunPCA” method in the Seurat package [25]. To perform an exact cellular clustering analysis, the top 20 main components were used. Gene expression was displayed using the “VizDimLoadings”, “DimPlot” and “DimHeatmap” functions for PCAs. Following that, cell clustering was identified using the “JackStraw, num.replicate = 100,” “ScoreJackStraw, dims = 1:20,” “JackStrawPlot,” “ElbowPlot” functions, “FindNeighbors, dims = 1:20,” and “FindClusters” functions in the Seurat package [25], with the criterion “resolution” set to 0.5. Furthermore, the uniform manifold approximation and projection (UMAP) approach using the “RunUMAP” function was used to reduce dimensionality and identify clusters. The “FindAllMarkers” function was employed to estimate the false discovery rates (Adj.Pvalue) and log2foldchange to identify the genes that had differential expression (DEGs) in each cluster. DEGs with logfc. threshold = 1 and min.pct = 0.25 were considered the marker gene for each cluster. Finally, the SingleR [26], celldex [26] and SingleCellExperiment [27] R packages were utilized to automatically cluster and annotate cell types utilizing the “HumanPrimaryCellAtlasData” function.

### 2.5 Mapping Single‐Cell Pseudotime Evolutionary Trajectories

Single‐cell trajectory evaluation was carried out using the R package Monocle2 (v2.30.0) to identify the cell state developments [28]. We imported RDS files such as cell types into R programming language. The “newCellDataSet” function was utilized to construct a new object with the input parameters “expressionFamily = negbinomial.size” and “lowerDetectionLimit = 0.5”. The parameters such as “reduction_method = “DDRTree” and “max_components = 2” were used to reduce dimensions using the “reduceDimension” function. The cell lineage trajectories were then determined employing pseudotime and cell clustering, utilizing the default parameters of the Monocle2 package [28]. The findings were then displayed using the “plot_cell_trajectory” function.

### 2.6 Decoding of Cell‐Cell Interaction Landscapes

The intercellular networks of interaction are extensively estimated from scRNA‐seq data using a new package called CellChat [29]. Using pattern identification algorithms and the human connection between ligand and receptor databases, CellChat (https://github.com/sqjin/CellChat) can anticipate the primary signaling inputs and outputs for cells and discover how these cells and signals communicate with one another [29]. We discovered possible cell‐cell interactions for two groups: healthy‐mild and healthy‐severe. A comprehensive database encompassing secreted signaling, ECM‐receptor and cell‐cell interaction connections provided a framework for CellChat. We used the R programming language to load the RDS data, including normalized count and cell types, from the Seurat package [25] into CellChat (v2.1.2) [29] in accordance with the instructions. Processing algorithms including “identifyOverExpressedGenes” and “identifyOverExpressedInteractions” with normal parameters were employed. Important functions such as “aggregateNet,” “computeCommunProb” and “computeCommunProbPathway” were utilized for the main study based on general criteria. Connections were displayed utilizing the R programming language.

### 2.7 GraphST Hyperparameter Settings, Training Monitoring, and Validation Strategy

GraphST [30] was trained with 1200 epochs and a fixed random seed of 50 to ensure reproducibility. Model training was executed on GPU (CUDA‐enabled) when available, or CPU otherwise. To ensure convergence, training loss was visually monitored and stabilized around epochs 1000–1100, indicating adequate model fitting. Before training, we used the “GraphST.preprocess.filter_with_overlap_gene” function to retain highly variable and shared genes between the spatial and scRNA‐seq datasets, reducing feature mismatch and improving integration quality. Model performance was assessed via three complementary approaches: (1) biological consistency of predicted immune cell distribution across lung tissue sections, (2) agreement between spatial cell‐type localization and viral gene expression levels, and (3) preservation of known immunological structures such as increased neutrophil and monocyte presence in infected regions. These steps ensured the interpretability and robustness of GraphST‐based spatial deconvolution. To quantify spatial patterns and validate the nonrandom distribution of gene expression, we performed spatial statistical analyses. Specifically, we calculated Moran's I to measure spatial autocorrelation and detect gene expression clustering. These analyses provided quantitative support for the observed spatial heterogeneity and were implemented using the esda [31] and libpysal [31] Python packages.

### 2.8 Exploration of Spatial Gene Expression and Deconvolution

We used a complete set of packages to improve the computing, processing and visualization components of our findings for the spatial evaluation. These consisted of GraphST [30] for graph‐based spatial assessment, OS for management of files, Torch [32] for deep learning assistance, Skmisc [33] and Scikit‐learn to perform extra statistical and metric calculations, OT [34] for optimal transport evaluation, Matplotlib [35] and its submodule Pyplot for graphical representation, Pandas [36] and NumPy [37] for data manipulation and Scanpy [38] and AnnData for conducting spatial transcriptomics data. Employing the GraphST [30] package in a Python environment, we merged single‐cell RNA seq (scRNA‐seq) and spatial transcriptomics data to explain the spatial arrangement and cellular composition of lung tissues (COVID‐19 and healthy). The R packages SeuratData [39] and SeuratDisk [40] were used for converting the scRNA‐seq data, which had been initially recorded in the RDS format, into the AnnData‐compatible h5ad format. This conversion was facilitated by specifying the R programming language. Following that, the “scanpy.read_h5ad” function from the Scanpy [38] library was used to import the single‐cell data set into Python. The filtered feature‐barcode matrix (filtered_feature_bc_matrix.h5) and associated images with high resolution were also included in the spatial transcriptomics data that was loaded using “scanpy.read_visium” function from the Visium platform. Utilizing the “var_names_make_unique” technique, distinct names for variables were allocated to the single‐cell and spatial datasets to facilitate comparability and prevent naming conflicts. To improve the accuracy of the data and provide analytical reliability, both the spatial and single‐cell datasets completed a thorough preparation process. Employing “sc. pp. normalize_total” function for normalizing the spatial data set (called as “data_spatial”) with a target sum of 10,000 counts per spot, log‐transformation was subsequently carried out utilizing “sc.pp.log1p” to stabilize variance. “sc.pp.highly_variable_genes” function was employed to identify highly variable genes (HVGs). The top 2000 genes were chosen based on their dispersion, and the data set was then filtered to these genes. We used “GraphST.preprocess” function to prepare the spatial data for GraphST assessment, “GraphST. construct_interaction” function was used to create spatial connections to capture neighborhood interactions, and “GraphST.add_contrastive_label” function was utilized to add distinct labels to enhance feature discrimination. The single‐cell data set, known as “data_singlecell” underwent a similar processing procedure. “sc.pp.normalize_total(target_sum=1e4)” function was employed for normalization, while “sc.pp.log1p” function was applied for log‐transformation. “data_singlecell.X[data_singlecell.X < 0] = 0” function was used to set negative expression values to zero to address data objects, and “np.nan_to_num” with the parameters “nan=0.0”, “posinf=0.0”, and “neginf=0.0” was employed to substitute absent or infinity values with zeros. “sc.pp.filter_cells(min_genes=1)” function was utilized to filter cells expressing a minimum of one gene, and “sc.pp.filter_genes(min_cells=1)” function was used to filter genes expressed in a least one cell. The data set was filtered in accordance with the selection of HVGs using “sc.pp.highly_variable_genes(n_top_genes=2000)” function. The single‐cell data was then aligned using the spatial method employing the “GraphST.preprocess” function. The “GraphST.preprocess.filter_with_overlap_gene” function was used to balance the feature spaces of “data_spatial” and “data_singlecell” with the goal to identify and preserve overlapping genes and guarantee data set comparability. The “GraphST.get_feature” function was used to obtain features from the spatial data for model training and deconvolution, producing a representation appropriate for downstream analysis. GraphST was set up with 1,200 epochs, a random seed of 50 for reproducibility, and computation assigned to a CUDA‐capable GPU (‘torch.device(‘cuda:1’)‘) if it was obtainable, otherwise to the CPU. Using the single‐cell reference data, the “deconvolution=True” option was configured to allow an estimation of cell type compositions inside each spatial location. “model. train_map” function was used to train the model. It combined the single‐cell and spatial datasets by projecting cellular profiles into the spatial context, producing modified “data_spatial” and “data_singlecell” objects. The single‐cell data was annotated with cell types according to specified frequencies that were recorded in a list of terms (referred to as “frequencies”). The “data_singlecell. obs[‘cell_type’]” column was updated with a list of cell types (“cell_type_list”) that was created by duplicating every type of cell based on its frequency. “GraphST.utils.project_cell_to_spot” function was then used to project the single‐cell annotations into the spatial spots, keeping 15% of the top‐ranked features (“retain_percent=0.15”) for ensuring reliable deconvolution with reducing noise. We employed the “scanpy. pl. spatial” function from the Scanpy package to demonstrate the spatial distribution of deconvoluted cell types. This plotting function was applied to the “data_spatial” object, highlighting specific cell types of interest for healthy‐mild group such as: Neutrophil, Monocyte:CD14 + , T cell:CD4+ central memory, T cell:CD4+ Naive, NK cell, T cell:CD4+ effector memory, Platelets, B cell:immature, Pre‐B cell CD34‐ and T cell:CD8 + . For healthy‐severe group including: Neutrophil, Monocyte:CD14 + , T cell:CD4+ central memory, T cell:CD4+ Naive, T cell:CD4+ effector memory, NK cell, Platelets, B cell:Naive, T cell:gamma‐delta and Pre‐B cell CD34−. By mapping the spatial distribution of cells according to spatial coordinates, we were able to show the percentage of cells in various lung tissue locations using the magma and viridis color palettes for distribution of cells. magma and cividis color palettes were used for demonstrated of gene expression in lung tissues related to the COVID‐19 and healthy.

### 2.9 Data Collection for Mutation Analysis

We obtained permission from John A. Burns School of Medicine of University of Hawaii to access data from the GISAID database (https://gisaid.org/). All available SARS‐CoV‐2 full‐length genome sequences, along with amino acid sequences, geographic locations and sample submission dates, were downloaded in FASTA format by June 11, 2023. Initial processing involved the exclusion of divergent, short or lengthy sequences in comparison to the reference sequence (accession no. NC_045512.2), sequence gaps, unspecified amino acids (indicated by X) and samples originating from nonhuman hosts. This step aimed to eliminate irrelevant or low‐quality data before further analysis [41].

### 2.10 Sequence Alignment, Trimming, and Variant Calling

All the pre and post processing of FASTA files was done using Python programming language (Version 3.8.0). The remaining sequences were aligned to the reference SARS‐CoV‐2 genome from Wuhan using the EVcouplings [42] Python package. The 3ʹ and 5ʹ terminus regions were excluded after alignment to eliminate large numbers of missing and ambiguous reads and to achieve better alignment accuracy. This step facilitated the identification of variations and mutations in the sequences. After alignment, each high‐quality sequence was compared with the reference to identify variations and mutations. This process was essential for uncovering the amino acid alterations present in the virus samples. An analysis was conducted to determine the frequency of mutation incidence over time. This enabled the identification of persistent mutations across different samples and timelines. R (version 4.1.3) programming language and Microsoft Power BI were utilized for data standardization and comparison chart outlining. Each region's normalized frequency was used to compare the data from each continent properly. For this purpose, the number of mutations on each continent was divided by the number of similar sequences in equal proportions.

### 2.11 Mutation Tracking: Origin and Lineages Detection

After identifying persistent mutations based on their frequencies and timelines, we tracked the initial occurrence of each mutation in the data set. This step involved determining which sample first exhibited each sustainable mutation, allowing for the identification of the mutation's geographical origin. We used the https://www.ncbi.nlm.nih.gov/activ to determine the common lineage of each mutation based on timelines.

### 2.12 Structural Modeling of Proteins: Pre and Postmutation Analysis

We employed various structure‐based bioinformatics methods to determine the impact of the many significant mutations discovered in this study on the stability of SARS‐CoV‐2 proteins. To predict changes in stability due to mutations, various computational techniques were developed. We categorized the impact of each mutation on protein structure as stabilizing or destabilizing using methods such as Gibbs free energy values (ΔΔG), mutation Cutoff Scanning Matrix (ΔΔG mCSM), Site‐Directed Mutations (ΔΔG SDM), Distance Dependent Energetics based Unified Theory (ΔΔG DUET) and Elastic Network Contact Model (ΔΔG ENCoM and ΔΔS ENCoM). DynaMut (https://biosig.lab.uq.edu.au/dynamut/) [43] and DynaMut2 (https://biosig.lab.uq.edu.au/dynamut2/) [44] were used to predict the effects of missense mutations on protein stability. Protein Data Bank (PDB) (https://www.rcsb.org/) [45] was used to retrieve the wild‐type proteins of SARS‐CoV‐2's crystal structures. A primary limitation of this study was the lack of wild‐type PDB structures for specific proteins (NSP4, NSP6, ORF6, and ORF9c), crucial for understanding the effects of mutations on protein conformation. Although some structures were available, the majority did not represent the reference genome protein and had inherent mutations.

### 2.13 Statistical Analysis

We used standard and widely adopted computational tools including Seurat for data processing, SingleR and Celldex for cell type annotation, Monocle2 for trajectory analysis, CellChat for cell–cell communication, and GraphST for spatial pattern detection. R programming language (v4.3.2, R Foundation for Statistical Computing, Vienna, Austria; http://www.r-project.org/) and Python was used for statistical analysis and data visualization including Seurat, celldex, SingleCellExperiment, SingleR, Monocle2, CellChat, GraphST, EVcouplings, Scanpy, Matplotlib, OS, Torch, Pandas, NumPy, Skmisc, Scikit‐learn, OT. A p‐value of 0.05 or lower was considered to be the significance threshold for this statistical study.

## 3 Results

### 3.1 Single Cell Transcriptome Analysis of Healthy, Mild, and Severe Blood Samples

By employing the Seurat R package, we performed a scRNA‐seq assessment of 12 blood samples, including 4 healthy samples (21 294 genes, 23 389 cells), 4 mild samples (21 376 genes, 17 025 cells) and 4 severe samples (20 349 genes, 14 229 cells). After normalizing scRNA‐seq data and evaluating quality, lower‐quality cells were subsequently removed with options: Only cells expressed between 200 and 5000 genes for healthy and severe samples, and between 200 and 4500 genes for mild samples, as well as genes expressed in at least 3 cells, were selected. Subsequently for additional analysis, the top 2000 genes with the largest variation were selected. After that, we investigated reductions in dimension between samples, such as healthy‐mild and healthy‐severe. We utilized the RunPCA function to reduce the PCA dimensions of the top 2000 highly variable genes. Numerous “important” PCs with low p values were discovered. The JackStrawPlot approach was used to demonstrate the highest 20 main components. The variance for every PC was analyzed and compared to the median dispersion. The data of highly diverse genes was fully reflected by the “important” PCs, which typically had a low p‐value. While utilized with the ElbowPlot function, it was found that, even though the bending demonstrates occurred approximately the 10th PC, the change decreased gradually after the 20th. Thus, we generated a heatmap of the original PCs, emphasizing their most significant genes to demonstrate how their DEGs differentiated compared to those of other PCs. Ultimately, we selected the top 20 PCs for additional UMAP analysis.

### 3.2 Cell Typing Between Healthy and Mild Samples

**Figure 2:**
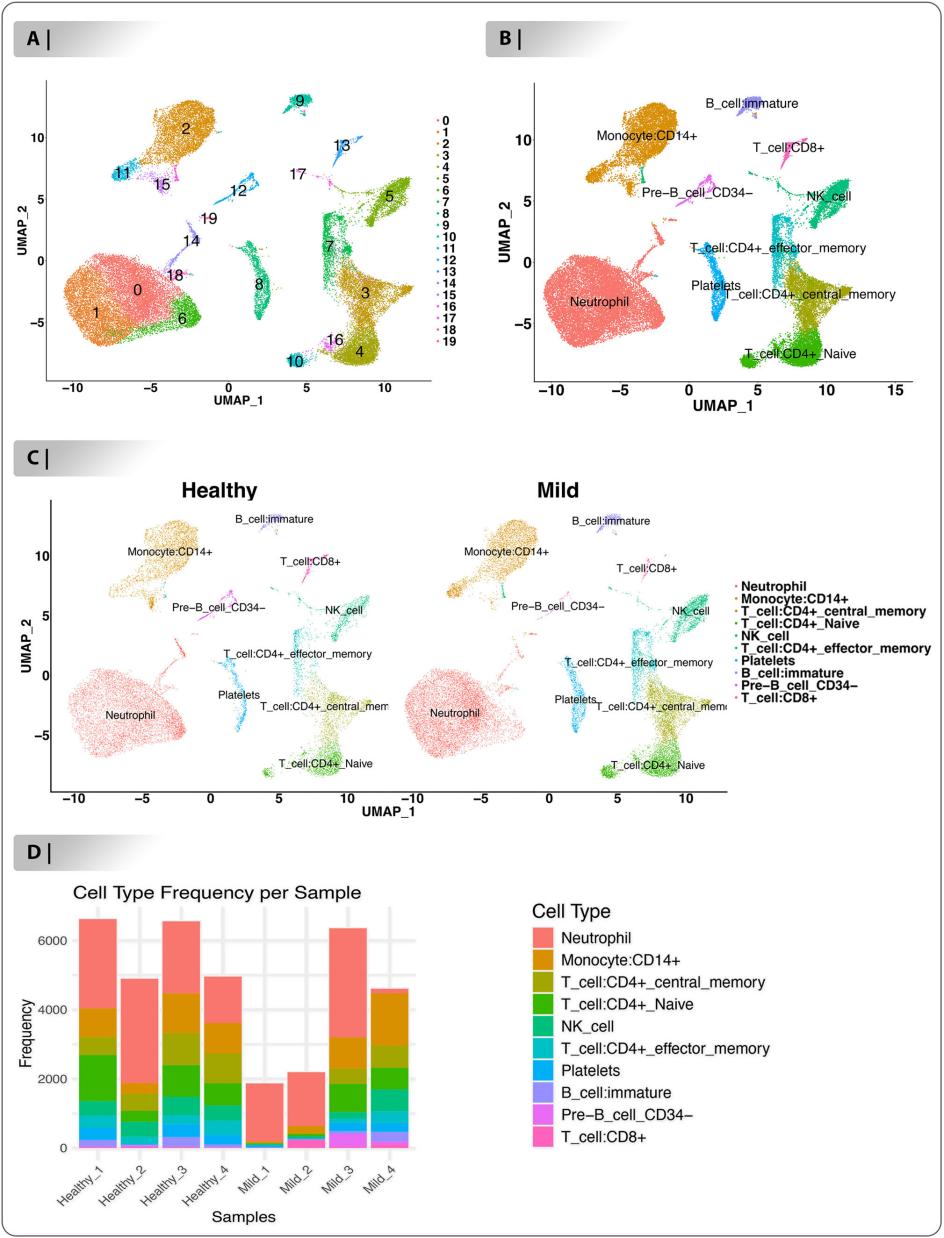
The characteristics of different cell groups between healthy‐mild blood samples. (A). The umap plot of integrated samples such as: four healthy samples (21 294 genes and 23 389) and four mild samples (21 376 genes, 17 025 cells). (B). 20 cluster annotated to the 10 cell types including: Neutrophil, Monocyte:CD14 + , T cell:CD4+ central memory, T cell:CD4+ Naive, NK cell, T cell:CD4+ effector memory, Platelets, B cell:immature, Pre‐B cell CD34‐ and T cell:CD8 + . (C). Proportions of cells population in two class between healthy and mild samples. (D). Proportions of cells population in each sample of healthy and mild.

With the goal to investigate the properties of various cell groups between healthy and mild, samples were merged together as indicated by the umap plot (Figure 2A). Furthermore, cells were clustered utilizing the “FindCluster” function to generate 20 clusters (Figure 2A). Following that, we identified 10 different cell types within these clusters using SingleR package, a computational annotation tool based on the Human Primary Cell Atlas database. Notable cell types included: Neutrophil (cluster annotated: C0, C1, C6, C14, C18, and C19), Monocyte:CD14+ (cluster annotated: C2, C11, and C15), T cell:CD4+ central memory (cluster annotated: C3), T cell:CD4+ Naive (cluster annotated: C4, C10 and C16), NK cell (cluster annotated: C5 and C17), T cell:CD4+ effector memory (cluster annotated: C7), Platelets (cluster annotated: C8), B cell:immature (cluster annotated: C9), Pre‐B cell CD34‐ (cluster annotated: C12) and T cell:CD8+ (cluster annotated: C13) (Figure 2B). Depicting that Neutrophil, Monocyte:CD14+ and T cell:CD4+ central memory are the most predominant cell types in healthy and mild samples (Figure 2B). Subsequently, comparing the proportions of each population in the healthy and mild samples, it was found that in the immune cell population, the proportion of T cell:CD4+ central memory (healthy vs. mild: 7.51% vs. 12.18%), T cell:CD4+ Naive (healthy vs. mild: 10.12% vs. 13.92%), NK cell (healthy vs. mild: 6.03% vs. 7.90%), T cell:CD4+ effector memory (healthy vs. mild: 3.15% vs. 5.47%), Platelets (healthy vs. mild: 3.88% vs. 4.32%), B cell:immature (healthy vs. mild: 2.34% vs. 2.48%), in the mild blood samples was significantly increased, while the abundance of Neutrophil (healthy vs. mild: 43.32% vs. 39.17%), Monocyte:CD14+ (healthy vs. mild: 17.85% vs. 13.78%), Pre‐B cell CD34‐ (healthy vs. mild: 3.33% vs. 0.29%) and T cell:CD8+ (healthy vs. mild: 2.42% vs. 0.45%) was significantly decreased (Figure 2C, Table S1). Following that, as illustrated in Figure 2D, we evaluated the percentage of 10 cell types in mild and healthy blood samples.

### 3.3 Cell Typing Between Healthy and Severe Samples

**Figure 3:**
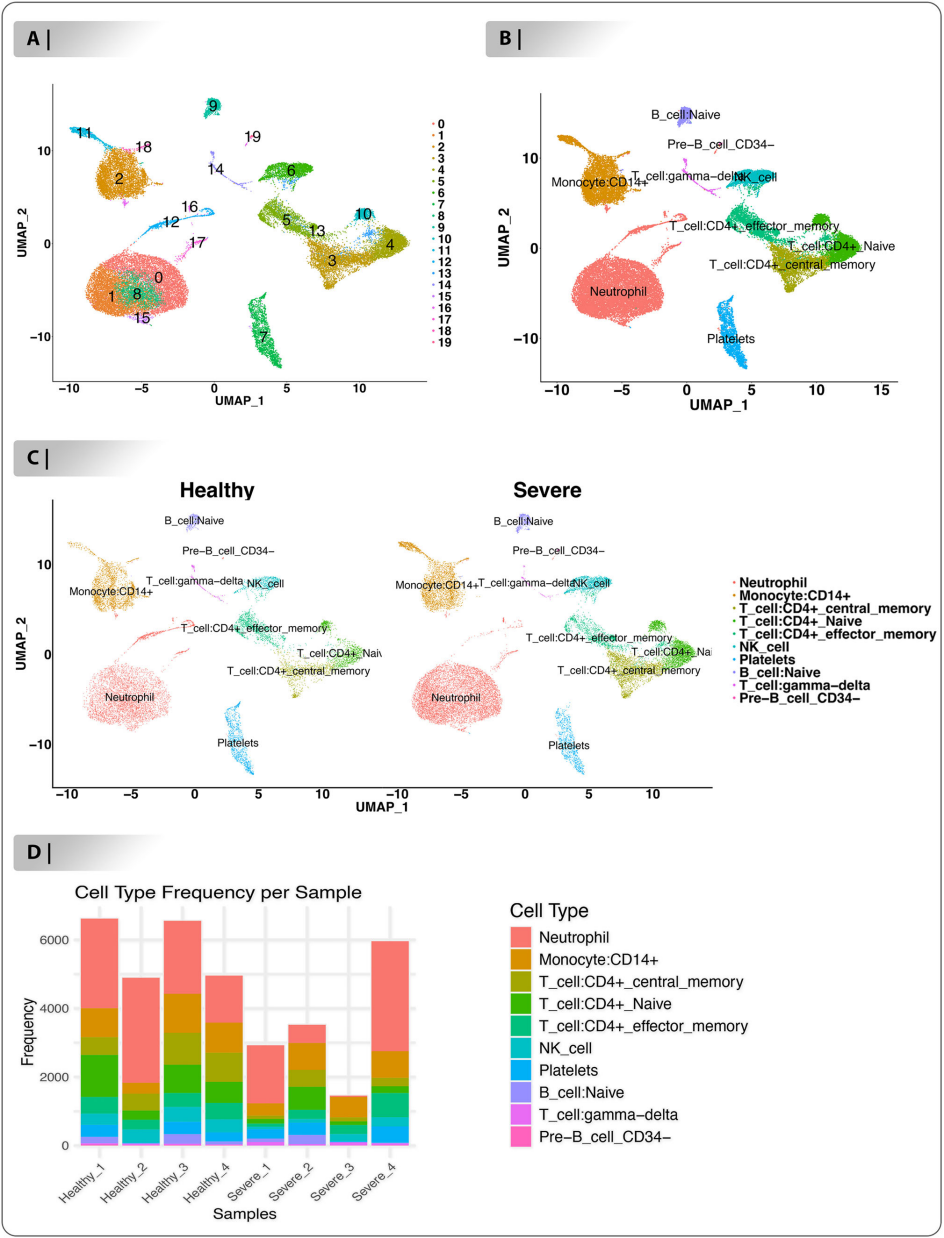
The characteristics of different cell groups between healthy‐severe blood samples. (A). The umap plot of integrated samples such as: four healthy samples (21 294 genes and 23 389) and 4 severe samples (20 349 genes, 14 229 cells). (B). 20 cluster annotated to the 10 cell types including: Neutrophil, Monocyte:CD14 + , T cell:CD4+ central memory, T cell:CD4+ Naive, T cell:CD4+ effector memory, NK cell, Platelets, B cell:Naive, T cell:gamma‐delta and Pre‐B cell CD34‐. (C). Proportions of cells population in two class between healthy and severe samples. (D). Proportions of cells population in each sample of healthy and severe.

The umap plot shows that many cell clusters among healthy and severe were identified through integrating the samples (Figure 3A). Additionally, 20 clusters were created by clustering cells employing the “FindCluster” function (Figure 3A). Next, utilizing SingleR package, a computational annotation program based on the Human Primary Cell Atlas database, we were able to identify 10 distinct cell types employing these clusters. These cell types consisted of: Neutrophil (Cluster annotated: C0, C1, C8, C12, C15, C16, and C17), Monocyte:CD14+ (Cluster annotated: C2, C11, and C18), T cell:CD4+ central memory (Cluster annotated: C3), T cell:CD4+ Naive (Cluster annotated: C4 and C10), T cell:CD4+ effector memory (Cluster annotated: C5 and C13), NK cell (Cluster annotated: C6), Platelets (Cluster annotated: C7), B cell:Naive (Cluster annotated: C9), T cell:gamma‐delta (Cluster annotated: C14) and Pre‐B cell CD34‐ (Cluster annotated: C19), demonstrated that the most common cell types in healthy and severe samples are Neutrophil, Monocyte:CD14+ and T cell:CD4+ central memory (Figure 3B). After comparing the percentages of each population in the healthy and severe samples, it was determined that within the immune cell population, the percentage of Neutrophil (Healthy vs. severe: 39.45% vs. 39.85%), T cell:CD4+ central memory (Healthy vs. severe: 6.90% vs. 12.11%), T cell:CD4+ Naive (Healthy vs. severe: 8.02% vs. 12.75%), NK cell (Healthy vs. severe: 4.72% vs. 6.63%) and Pre‐B cell CD34‐ (Healthy vs. severe: 0.13% vs. 0.27%) in the severe blood samples was significantly increased, while the proportion of Monocyte:CD14+ (Healthy vs. severe: 18.13% vs. 13.80%), T cell:CD4+ effector memory (Healthy vs. severe: 9.56% vs. 7.17%), Platelets (Healthy vs. severe: 8.20% vs. 4.31%), B cell:Naive (Healthy vs. severe: 2.79% vs. 2.48%), T cell:gamma‐delta (Healthy vs. severe: 2.03% vs. 0.58%) was significantly reduced (Figure 3C, Table S1). Next, the proportion of every one of the 10 cell types in both healthy and severe blood samples was investigated and shown in (Figure 3D).

### 3.4 Development of Pseudotime Trajectories Between Healthy‐Mild and Healthy‐Severe

**Figure 4:**
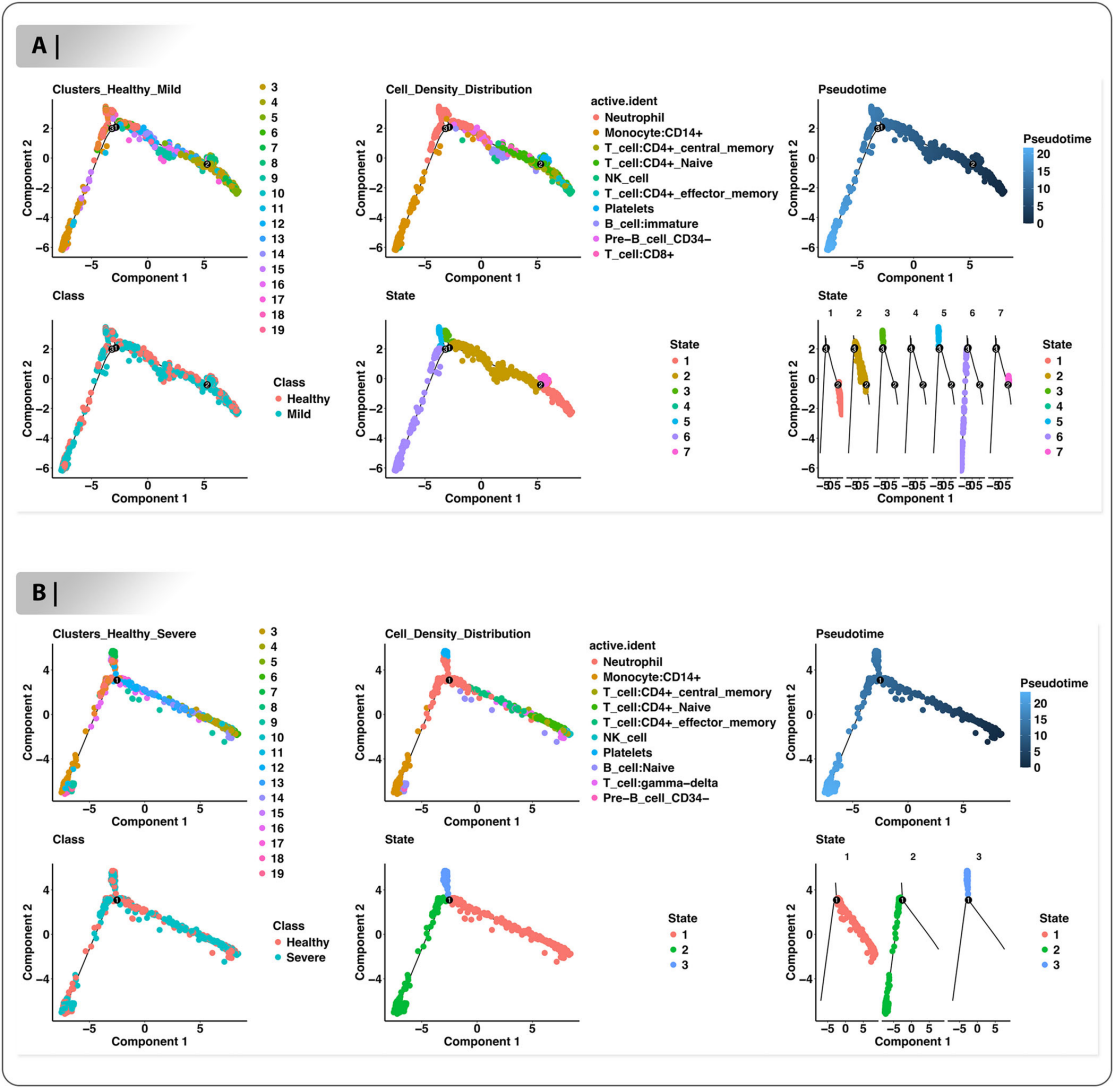
Simulation of the trajectory of blood cells development determined by Monocle2 algorithm. Pseudotime trajectory of (A) healthy‐mild and (B) healthy‐severe groups based on the cell clusters, cell source transition, pseudotime, class and cell type transition.

To investigate the immune cell differentiation and activation dynamics in COVID‐19, we applied Monocle2 pseudotime analysis to peripheral blood samples from mild and severe cases. The trajectory for the healthy–mild comparison showed a branching pattern composed of one pre‐branch and two main trajectories, totaling seven pseudo temporal states (Figure 4A). The root state was defined based on the initial cluster enriched with Neutrophils and Monocytes:CD14 + , reflecting their role in the early innate response (Figure 4A). As pseudotime progressed, T cell:CD4+ Naive, NK cells, and Pre‐B cell CD34‐ emerged toward the terminal ends of separate branches, indicating their activation or transition later in the immune response (Figure 4A). In the healthy–severe comparison, the trajectory revealed a three‐state progression with Neutrophils and Platelets dominating the early (root) state (Figure 4B). The two diverging branches captured distinct differentiation paths: one culminating in T cell:CD4+ Naive, T cell:gamma‐delta, and NK cells, and the other ending in Monocyte:CD14 + , Pre‐B cell CD34‐, and B cell:Naive (Figure 4B). These results suggest a coordinated evolution from innate to adaptive immunity, with lineage trajectories diverging toward effector populations depending on disease severity. The root state assignment and branch‐specific cell populations provide biologically meaningful insights into immune progression during SARS‐CoV‐2 infection.

### 3.5 Communication Networks Between Cells

**Figure 5:**
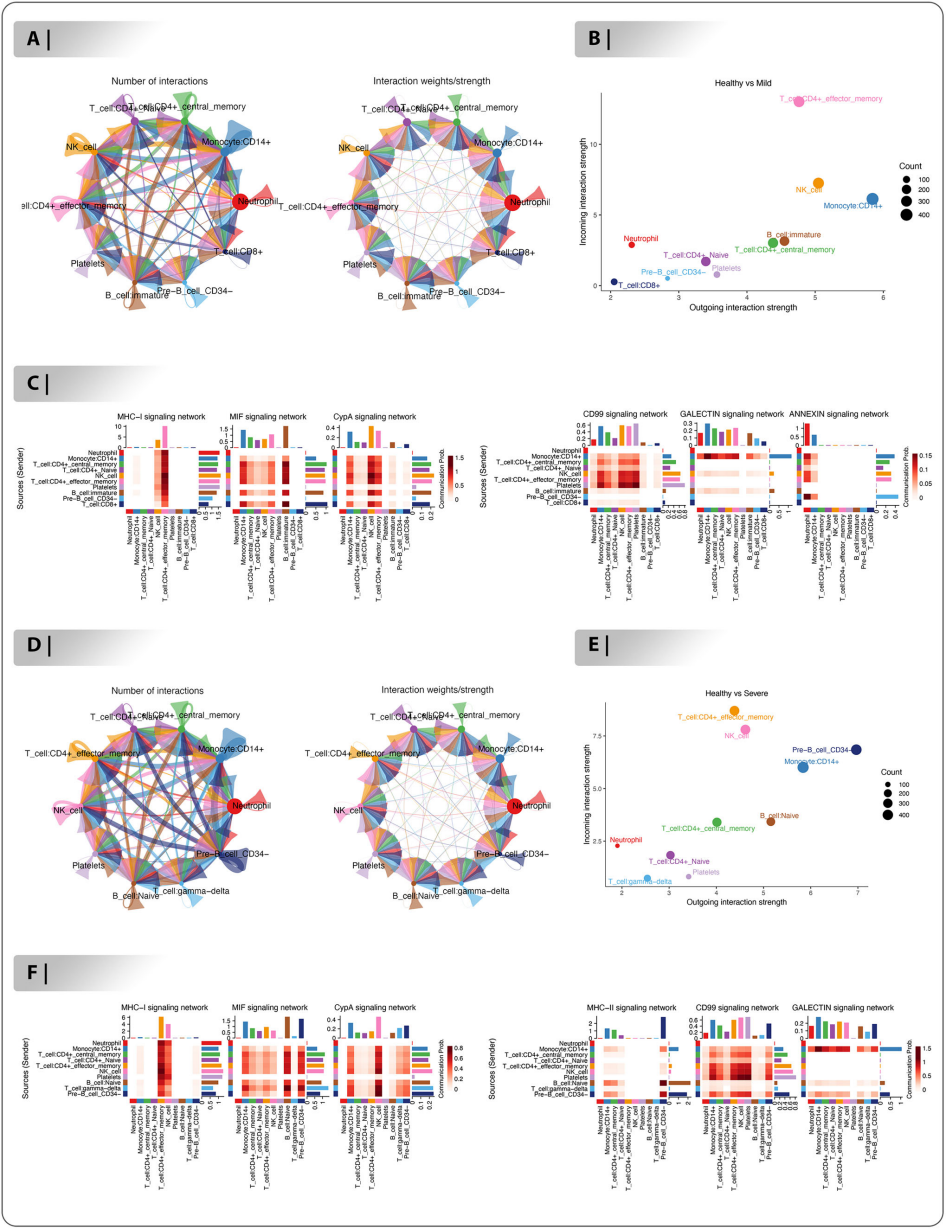
Communication among cell types in COVID‐19 samples. (A). Cell‐cell communication signaling pathway networks between immune cell types using cell chat algorithm in healthy‐mild. The interaction strength appears on the right, while the interaction number is represented on the left. (B). The incoming and outgoing strength of cell types between healthy‐mild. (C). The immune‐related signaling pathway networks are represented in the netVisual heatmap such as: MHC‐I, MIF, CypA, CD99, GALECTIN and ANNEXIN signaling pathways for healthy‐mild group. (D). Complexes of signaling pathways for cell‐cell communication amongst immune cell types in healthy‐severe. (E). The incoming and outgoing strength of cell types between healthy‐severe. (F). The immune‐related signaling pathway networks are demonstrated in the netVisual heatmap including: MHC‐I, MIF, CypA, MHC‐II, CD99 and GALECTIN signaling pathways for healthy‐severe group.

We have investigated the network of cell‐cell interactions between the two groups, containing healthy‐mild and healthy‐severe. This approach maps the expression of ligand‐receptor pairs among numerous immune cells in TME, providing for the identification of potential connections within cell types. In the healthy‐mild group, cellchat evaluation indicated that Monocyte:CD14+, T cell:CD4+ central memory and B cell:immature shown the strongest signals output capability (Figure 5A). Furthermore, the strongest incoming and outgoing signaling were determined by T cell:CD4+ effector memory and NK cells (Figure 5B). Focusing on MHC‐I, MIF, CypA, CD99, GALECTIN and ANNEXIN signaling pathways, we identified key immune cell types, including Neutrophils, Monocytes (CD14+), T‐cells (CD4+ central memory, naive, effector memory, CD8+), NK cells, B‐cells (immature, Pre‐B CD34‐) and Platelets, as major contributors to signaling networks (Figure 5C). In the MHC‐I signaling network, T cell:CD4+ effector memory exhibited the highest signaling strength, followed by NK cell, with strong communication probabilities with Platelets and Neutrophil, indicating robust antigen presentation and adaptive immune activation (Figure 5C). The MIF signaling network revealed elevated activity in B cell:immature, with significant interactions with T cell:CD8 + , T cell:CD4+ central memory and T cell:CD4+ effector memory, highlighting their role in driving pro‐inflammatory responses during infection (Figure 5C). In the CD99 signaling network, Platelets exhibited the highest signaling strength, followed by NK cell, with strong communication probabilities with Monocyte:CD14+ and T cell:CD4+ effector memory, suggesting their role in immune cell recruitment and coordination (Figure 5C). In the healthy‐severe group, cellchat analysis revealed that Pre‐B cell CD34‐ demonstrated the highest output capabilities for signals (Figure 5D). The strongest both incoming and outgoing signals were subsequently identified by Pre‐B cell CD34‐ and Monocyte:CD14+ (Figure 5E). Focusing on MHC‐I, MIF, CypA, MHC − II, CD99 and GALECTIN signaling pathways, we identified key immune cell types, including Neutrophils, Monocyte:CD14+ , T‐cells (CD4+ central memory, naive, effector memory, gamma−delta), NK cells, B‐cells (Naive, Pre‐B CD34‐) and Platelets, as major contributors to signaling networks (Figure 5F). The MHC−I signaling network revealed elevated activity in T cell:CD4+ effector memory, with significant interactions with NK cell, Neutrophil and Platelets, highlighting their role in driving pro‐inflammatory responses during infection (Figure 5F). The CD99 signaling network demonstrated increased activity in NK cell and Platelets, with significant connection with T cell:CD4+ effector memory, T cell:CD4+ central memory and Pre‐B cell CD34‐, emphasizing their capacity to contribute to inflammatory mediators' processes after infection (Figure 5F).

### 3.6 Spatial Distribution of Immune Cell Types in COVID‐19‐Affected Lung Tissue

**Figure 6:**
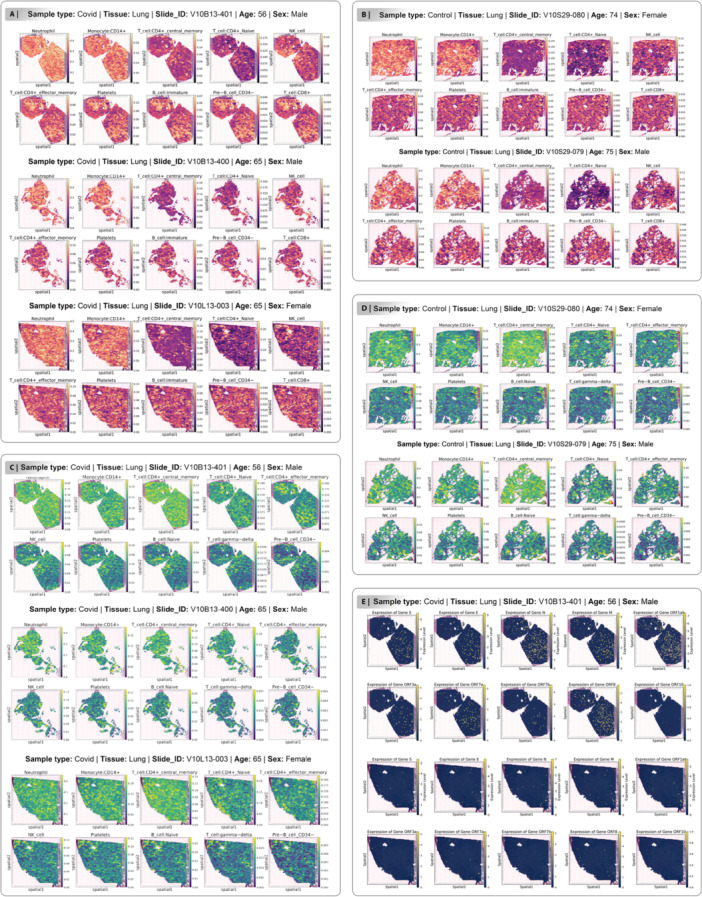
Spatial transcriptomics analysis of cell type distribution in COVID‐19 and control samples such as: V10B13‐401, V10B13‐400, V10L13‐003, V10S29‐080 and V10S29‐079. Each plot represents the abundance of a specific cell types in (A) COVID‐19 samples and (B) control samples for healthy‐mild group: Neutrophil, Monocyte:CD14 + , T cell:CD4+ central memory, T cell:CD4+ Naive, NK cell, T cell:CD4+ effector memory, Platelets, B cell:immature, Pre‐B cell CD34‐ and T cell:CD8 + , with the color gradient (magma colormap) indicating expression levels from low (purple) to high (yellow). (C) COVID‐19 samples and (D) control samples for healthy‐severe group: Neutrophil, Monocyte:CD14 + , T cell:CD4+ central memory, T cell:CD4+ Naive, T cell:CD4+ effector memory, NK cell, Platelets, B cell:Naive, T cell:gamma‐delta and Pre‐B cell CD34‐, with the color gradient (viridis colormap) indicating expression levels from low (green) to high (yellow). Spatial coordinates (spatial1 and spatial2) are shown on the x‐ and y‐axes, respectively, reflecting the anatomical organization of the tissue. (E) Spatial expression levels of viral genes (S, E, N, M, ORF1ab, ORF3a, ORF7a, ORF7b, ORF8 and ORF10) in lung tissues affected to the COVID‐19 in two samples such as: V10B13‐401 and V10L13‐003.

To investigate the molecular and cellular impacts of SARS‐CoV‐2 infection, we analyzed lung tissue samples from COVID‐19 patients and healthy using spatial transcriptomics. The data set comprised five samples: three COVID‐19 cases such as: V10B13‐401 (Age: 56, male), V10B13‐400 (Age: 65, male) and V10L13‐003 (Age: 65, female) and two healthy tissues including: V10S29‐080 (Age: 74, female) and V10S29‐079 (Age: 75, male). This selection of samples enabled a comparative analysis of gene expression profiles and cellular composition in infected and noninfected lung tissues, providing insights into the age‐ and sex‐specific effects of SARS‐CoV‐2 on pulmonary pathology. Spatial transcriptomics analysis of lung tissue from COVID‐19 patients in healthy‐mild group highlights the distribution and potential roles of distinct immune cell types, including Neutrophils, Monocyte:CD14+ and T cell:CD4+ central memory (Figure 6A). These cell types exhibit shared spatial characteristics, reflecting their involvement in the immune response to SARS‐CoV‐2 infection. Neutrophils, known for their role as first responders in innate immunity, show high density in central and upper regions of the tissue, indicative of their recruitment to sites of acute inflammation (Figure 6A). Similarly, Monocyte:CD14 + , which differentiate into macrophages and contribute to cytokine production and phagocytosis, are concentrated in overlapping regions, suggesting their participation in the inflammatory cascade (Figure 6A). T cell:CD4+ central memory, critical for adaptive immunity and long‐term immune memory, also cluster in these high‐density areas, potentially coordinating with innate immune cells to mount a sustained response against the virus (Figure 6A). Neutrophils and Monocyte:CD14+ exhibited high abundance, particularly in the central and upper‐left regions, suggesting localized immune activation in response to infection, while T‐cells, NK cells, and B‐cells showed lower but consistent presence across the tissue (Figure 6B). Spatial transcriptomics analysis of lung tissue from a COVID‐19 patient in healthy‐severe group revealed a high abundance of Neutrophils across three samples (Figure 6C). Neutrophils were predominantly localized in the central and upper‐left regions of the tissue (Figure 6C). Monocyte:CD14+ were primarily concentrated in the central regions of the tissue (Figure 6C). The consistent localization across replicates highlights the reliability of Monocyte:CD14+ as key players in the tissue's immune landscape. This distribution suggests a significant infiltration of Neutrophils and Monocyte:CD14+ in response to SARS‐CoV‐2 infection, likely contributing to localized inflammation in these areas. The consistency of Neutrophil localization across replicates underscores their critical role in the immune response within infected lung tissue. Neutrophils, Monocyte:CD14+ and T cell:CD4+ central memory consistently show the highest abundance, while Pre‐B cell CD34‐, T cell:gamma‐delta, B cell:Naive exhibit the lowest abundance (Figure 6D). To investigate the spatial expression of SARS‐CoV‐2 viral genes in infected lung tissue, we performed spatial transcriptomics analysis on a sample from a COVID‐19 patient (V10B13‐400, V10B13‐401 and V10L13‐003), focusing on 10 genes: S, E, N, M, ORF1ab, ORF3a, ORF7a, ORF7b, ORF8, and ORF10. In the V10B13‐401 sample, our result revealed that distinct expression patterns, with genes N and ORF1ab exhibiting the highest levels, predominantly in the central and upper‐right regions, indicative of active viral replication in these areas (Figure 6E). Genes S, ORF8 and M showed moderate expression, primarily in central regions, while genes E, ORF3a, ORF7a and ORF7b displayed low expression and ORF10 showed negligible expression (Figure 6E). These findings highlight the heterogeneous distribution of viral gene expression in COVID‐19‐affected lung tissue, providing insights into regional viral activity and its potential correlation with immune responses and tissue pathology. Our findings for V10L13‐003 sample, demonstrated that low expression for genes such as: S, E, N, M, ORF1ab, ORF7a and ORF8 in central regions (Figure 6E).

### 3.7 Spatial Correlation Between Immune Cell Abundance and Viral Gene Expression in COVID‐19 Lung Tissues

To investigate the relationship between immune cell localization and viral burden, we assessed the spatial co‐occurrence of dominant immune cell types and highly expressed viral genes in lung tissue samples from both mild and severe COVID‐19 groups. In the mild group, regions with elevated expression of viral genes such as: Spike, Envelope, Nucleoprotein, Membrane, ORF1ab, ORF7a, and ORF8 were colocalized with increased abundance of immune cell types including Neutrophils, Monocyte:CD14+, T cell:CD4+ central memory, T cell:CD4+ Naive, NK cell, Platelets, and Pre‐B cell CD34‐ (Figure 6E). Similarly, in the severe group, the expression of the same set of viral genes including: Spike, Envelope, Nucleoprotein, Membrane, ORF1ab, ORF7a, and ORF8 were showed a strong spatial association with immune cell types such as Neutrophils, Monocyte:CD14+, T cell:CD4+ central memory, T cell:CD4+ Naive, T cell:CD4+ effector memory, Platelets, and T cell:gamma‐delta (Figure 6E). These results suggest that immune cell infiltration within infected lung regions is tightly related to the local expression of key viral genes and highlighting the dynamic interaction between viral replication and the host immune response at the tissue level. Furthermore, we investigated the spatial expression patterns of SARS‐CoV‐2 viral genes in lung tissue from a COVID‐19 patient (sample V10B13‐401) using Moran's I statistic to assess spatial autocorrelation. The results reveal significant spatial clustering for most viral genes, indicating that their expression is not randomly distributed but rather localized within specific regions of the tissue. Notably, genes including Spike (FDR: 0.054), Envelope (FDR: 0.009), Nucleoprotein (FDR: 0.009), Membrane (FDR: 0.009), ORF1ab (FDR: 0.009), ORF3a (FDR: 0.009), ORF7a (FDR: 0.009), and ORF8 (FDR: 0.009) showed statistically significant spatial autocorrelation. In contrast, ORF7b (FDR: 0.297) did not demonstrate a significant spatial pattern. Among the genes studied, ORF1ab presented the highest degree of spatial clustering, suggesting potential hotspots of viral activity (Figure 6E, Figure S1A). These findings underscore the heterogeneous and focal nature of viral gene expression in infected lung tissue, which may contribute to the understanding of COVID‐19 pathophysiology and inform therapeutic approaches. For the lung tissue sample V10L13‐003 from a COVID‐19 patient, spatial analysis of viral gene expression using Moran's I revealed variable patterns of spatial autocorrelation. The Nucleoprotein (FDR: 0.009), Membrane (FDR: 0.018), ORF1ab (FDR: 0.009), and ORF7a (FDR: 0.09) genes showed statistically significant spatial clustering, indicating nonrandom, localized expression within the tissue. However, other genes including Spike (FDR: 0.373), Envelope (FDR: 0.1845), ORF3a (FDR: 0.315), ORF7b (FDR: 0.325125), and ORF8 (FDR: 0.1818) did not exhibit significant spatial patterns (Figure 6E, Figure S1B). These findings suggest that while certain viral genes display distinct focal expression in this sample, others appear more diffusely distributed or randomly expressed across the lung tissue. This heterogeneity in spatial gene expression may reflect differences in viral replication or host response across infected regions.

### 3.8 Tracking of Mutations for All Genes Related to the SARS‐CoV‐2

**Figure 7:**
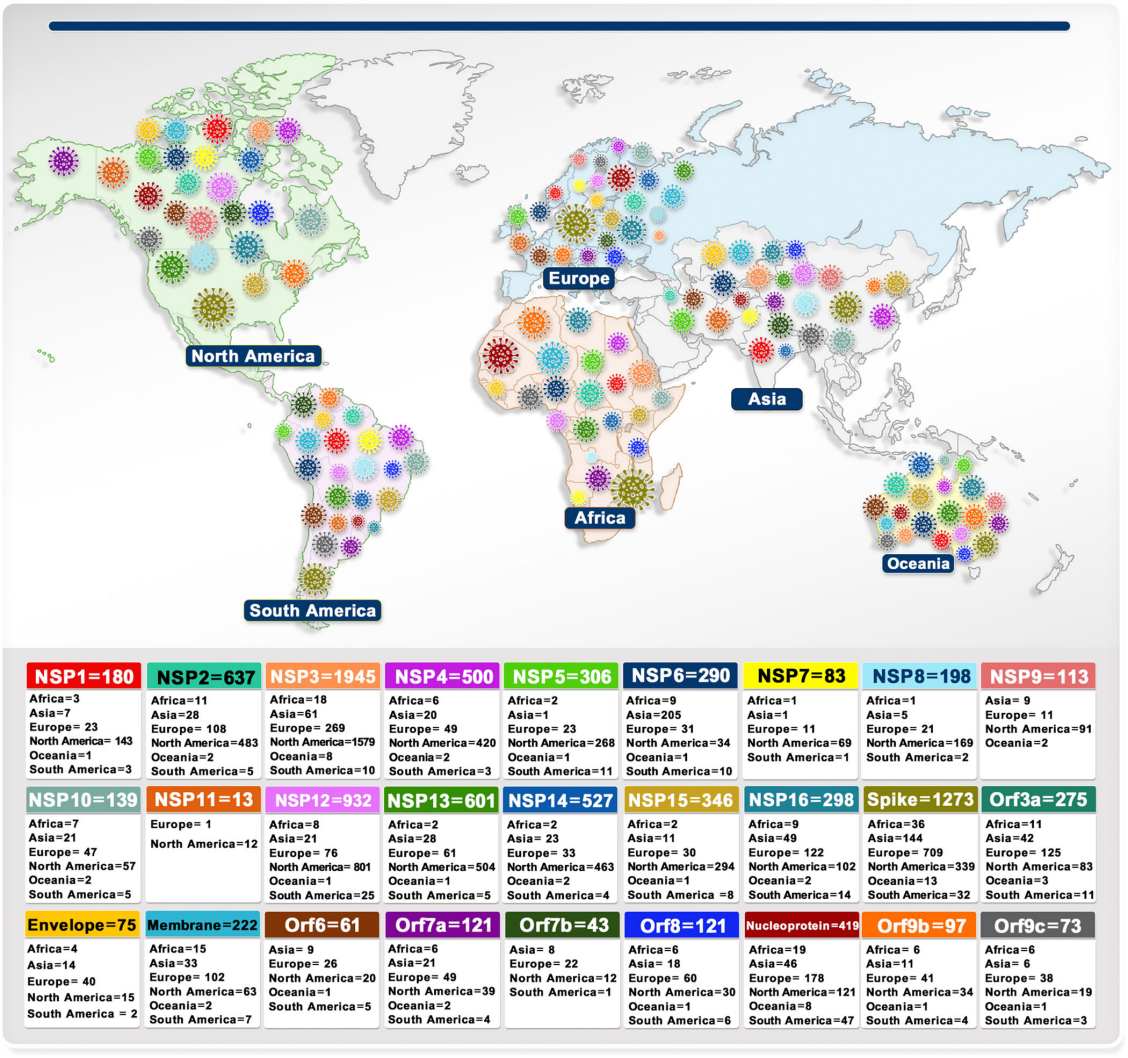
Number of detected mutations for genes in the continents of Africa, Asia, Europe, North America, Oceania and South America.

**Figure 8:**
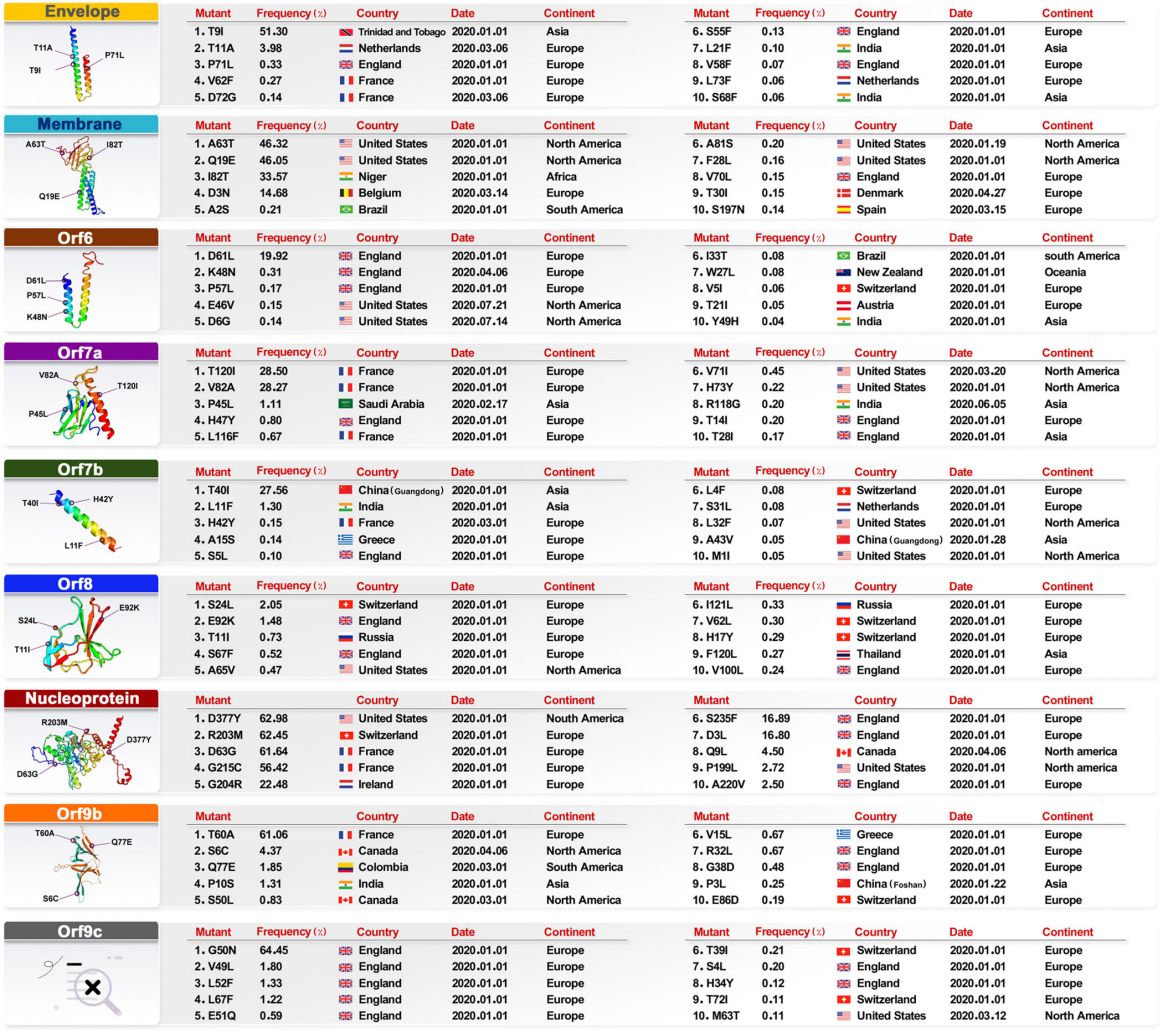
The top 10 mutations of genes (Envelope, Membrane, ORF6‐ORF8, Nucleoprotein, ORF9b‐ORF9c) such as: mutation position, mutation frequency, country name, mutation date and continent.

**Figure 9:**
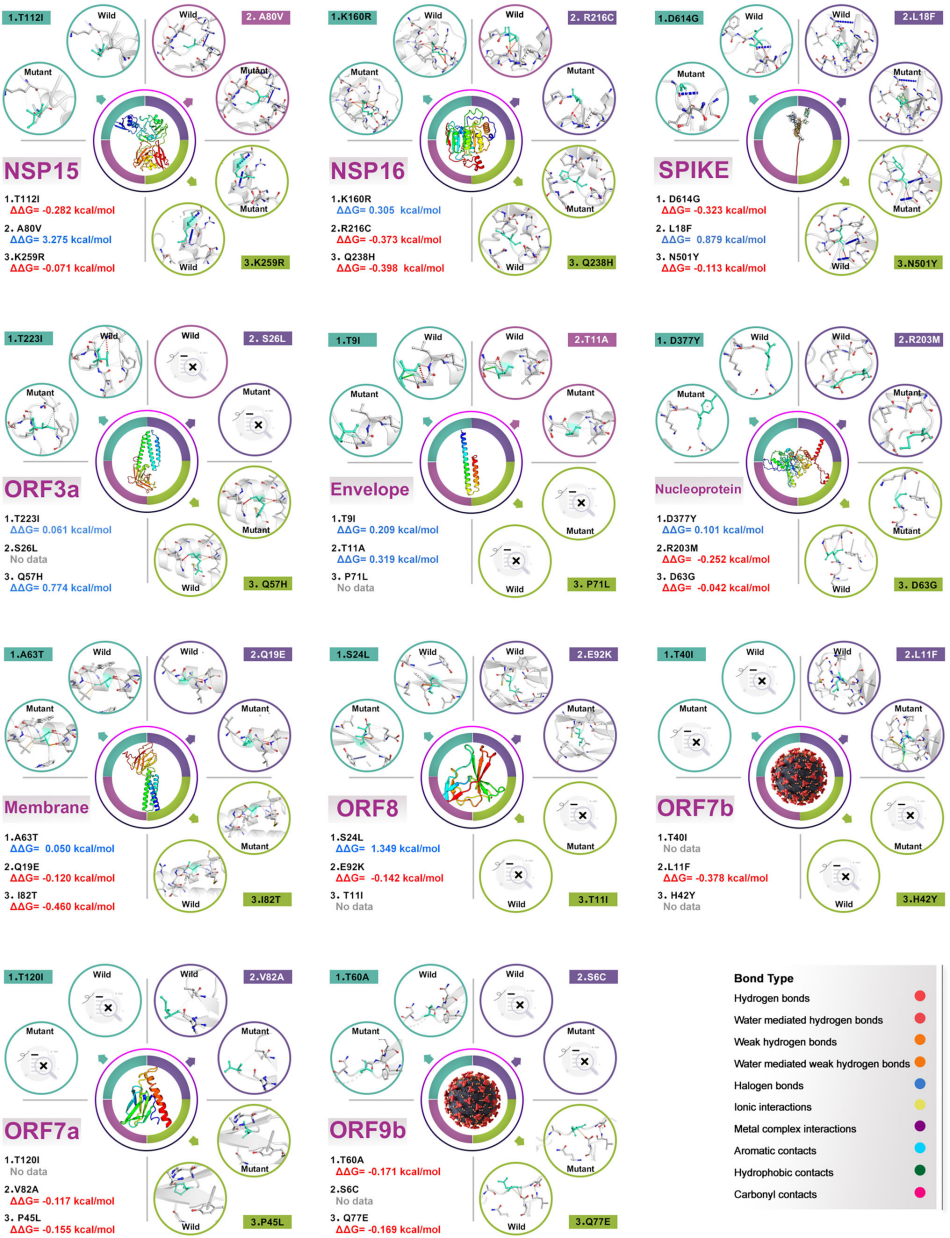
Results of the DynaMut server to investigate the effect of mutations on the stability or instability of the SARS‐CoV‐2 proteins including NSP15, NSP16, Spike, ORF3a, Envelope, Nucleoprotein, Membrane, ORF8, ORF7b, ORF7a and ORF9b.

According to the analysis of 15 669 529 samples, the sustainability mutations frequency has remained constant from January 1, 2020, to June 11, 2023. The result of NSP1 to NSP16, ORF6, ORF9b and ORF9c were presented in the Supporting Text. The number of all samples separately for each continent and the total number of samples for each gene are given in Table S2. All mutation frequency for each gene is categorized and represented in Table S3. The findings reported briefly explains the number of mutations in each continent (Figure 7), top three mutations with the highest frequency of each gene (Figure 8, Figure S2A,B) and most susceptible amino acids conversion in different positions and countries with different frequency shown in Table S5. To investigate the effect of mutations on the structure of proteins, we used the DynaMut online tool for protein modeling and calculated ΔΔG mCSM, ΔΔG SDM, ΔΔG DUET, ΔΔG ENCoM, and ΔΔS ENCoM, then subjected the final total to the recommended ΔΔG DynaMut to determine the top three mutations in each protein with the details reported in Figure 9, Figure S3 and Table S4. To effectively evaluate the mutation patterns of SARS‐CoV‐2 within China, it is crucial to consider the distinct geographical, demographic and epidemiological characteristics of key regions. Wuhan, Guangdong and Hong Kong have been selected as focal points for this analysis due to their significant roles in the early and ongoing phases of the pandemic, as well as their unique epidemiological profiles. Wuhan, As the initial epicenter of the SARS‐CoV‐2 outbreak. Wuhan provides critical insights into the virus's early mutations and spread. Studying this region helps us understand the origin and initial evolutionary dynamics of the virus. Guangdong Province, particularly the city of Guangzhou, has been a major hub of international trade and migration, making it a critical area for studying the introduction and spread of different viral strains. Its proximity to Hong Kong and the South China Sea also positions it as a significant area for monitoring cross‐border transmission and mutation patterns. Hong Kong, as a major global financial center with extensive international connections, has experienced multiple waves of SARS‐CoV‐2 infections. Its unique status as a special administrative region with a high population density and significant international travel provides valuable data on how mutations may spread in densely populated urban environments. By analyzing these regions, we can gain a comprehensive understanding of how different environments, population movements, and healthcare responses influence the evolution of SARS‐CoV‐2 within China. This regional approach also allows for targeted public health strategies to mitigate the spread of the virus and its variants.

### 3.9 Spike Mutation Patterns

The Spike protein is responsible for initiating an anchoring process with a receptor on the host cell surface, which is a necessary step before the merging of the viral membrane with the cell membrane. Additionally, this protein plays a key role in facilitating the viral entry process into the host cell. 1273 mutations were discovered in 83 countries which account for 709 mutations for Europe, 339 mutations for North America, 144 mutations for Asia, 36 mutations for Africa, 32 mutations for South America and 13 mutations for Oceania (Figure 7). D614G (XBB.1.5), the most common mutation in Spike, with a prevalence of 97.59% in Switzerland. The chemical spaces at position 614 is drastically changed by the mutation of Glycine (G), a little, nonpolar amino acid, to Aspartic Acid (D), a negatively charged, polar amino acid. While Glycine offers flexibility yet lacks the side chain required for powerful interactions, Aspartic Acid engages in hydrogen bonding and electrostatic interactions. This mutation most likely results in a decrease in electrostatic contacts, which causes instability and the D614G mutation is connected to greater viral infectivity despite the destabilizing effect because it improves spike protein flexibility and engagement with the ACE2 receptor. The frequency of L18F (XAY.1.1.1) in Switzerland is 15.91% in Europe. At position 18, stiffness can be added by replacing the small hydrophobic amino acid Leucine (L) with the bigger hydrophobic residue Phenylalanine (F), which has an aromatic ring. Combining interactions between the aromatic structure of Phenylalanine can improve the stability and wrapping in the hydrophobic core. Asparagine (N), a polar, uncharged amino acid, is mutated to Tyrosine (Y), an aromatic polar amino acid, introducing an aromatic ring and changing the hydrogen‐bonding potential at position 501. N501Y (XBB.1.5) has a frequency of 14.34% in Australia from Oceania. Tyrosine is capable of participating in the formation of hydrogen bonds and more stacking connections, which could improve binding affinity to the ACE2 receptor (Figure 8). The mutation of Acid Aspartic to Glycine in position 614 with ΔΔG ‐0.323 kcal/mol and mutation of Asparagine to Tyrosine in position 501 with ΔΔG −0.113 kcal/mol cause destabilization of the protein structure. The mutation of Leucine to Phenylalanine in position 18 with ΔΔG 0.879 kcal/mol cause stabilization of the spike protein structure (Figure 9, Table S4). 20 mutations in the conversion of Asparagine to Lysine (N>K) and 16 mutations in the conversion of Lysine to Asparagine (K>N) were found in spike protein. These mutations significantly changed the local charge distribution and electrostatic connections by shifting the position of an unaffected polar residue and a positively charged amino acid. Asparagine to Tyrosine (N>Y) and Tyrosine to Asparagine (Y>N) introduce or remove an aromatic ring, affecting the bonding of hydrogen and stacking potential, which could impact binding affinity and protein stability. Six mutations in the conversion of Tyrosine to Asparagine (Y>N) and six mutations in the conversion of Asparagine to Tyrosine (N>Y) are the amino acids that are most vulnerable to change in different places and nationality (Table S5).

### 3.10 Envelope Mutation Patterns

The heavy localization of the envelope has been observed at various intracellular transport sites, including viral assembly and budding at the endoplasmic reticulum (ER) and Golgi complex ERGIC. 75 mutations were found in 27 countries which account for 40 mutations for Europe, 15 mutations for North America, 14 mutations for Asia, 4 mutations for Africa and 2 mutations for South America (Figure 7). The largest frequency of mutations in the envelope, 51.30%, was identified in T9I (XBB.1.5) modifications discovered in Trinidad and Tobago, an Asian country. When Threonine (T), a polar amino acid with a hydroxyl group, is mutated to Isoleucine (I), a nonpolar, hydrophobic amino acid, a residue that can form hydrogen bonds is replaced with a larger, hydrophobic side chain. Because of this mutation, the protein is less able to make polar connections and more hydrophobic interactions take place, which probably helps to stabilize the envelope shape. The mutation T11A (XBB.1.5) in the envelope was found with a frequency of 3.98% in the Netherlands, Europe. It results from the replacement of the small, nonpolar amino acid Alanine (A) with the polar amino acid Threonine (T), which reduces the side chain and removes hydrogen bonding capability. This alteration most likely causes the envelope protein to pack better and to stabilize. With a frequency of 0.33%, the mutation P71L (BA.5.2) in the envelope was found in England, Europe. It results in an increase in conformational flexibility at position 71 by changing the rigid, cyclic amino acid Proline (P) to the flexible, hydrophobic residue Leucine (L). Leucine encourages the creation of secondary structures like helices, whereas Proline frequently causes structural splits. Probably, this modification enhances the region's stability and packaging (Figure 8). The mutation of Threonine to Isoleucine in position 9 with ΔΔG 0.209 kcal/mol and mutation of Threonine to Alanine in position 11 with ΔΔG 0.319 kcal/mol cause stabilization of the envelope structure (Figure 9, Table S4). We found that there are eight mutations in the conversion of Leucine to Phenylalanine (L>F) and five mutations in the conversion of Phenylalanine to Leucine (F>L). These mutations involve the substitution of two hydrophobic residues in the conversion of Leucine to Phenylalanine (L>F) and Phenylalanine to Leucine (F>L). However, the aromatic group of Phenylalanine adds inflexibility and the possibility of arranging interactions. Valine to Phenylalanine (V>F) conversion has four mutations that add comparable aromatic characteristics that can improve protein organizing in hydrophobic conditions or stabilize the protein core. These mutations take place in various locations and countries where the conversion of amino acids exhibits a high degree of vulnerability in envelope protein. These mutations most likely cause localized alterations in the stability of the envelope protein, especially in hydrophobic areas linked to viral assembly or membrane connection (Table S5).

### 3.11 Nucleoprotein Mutation Patterns

The Nucleocapsid Protein (N) is widely believed to possess a diverse array of functionalities, including the formation of helical ribonucleoprotein (RNP) complexes during the packaging of RNA genomes, as well as playing a crucial role in regulating viral RNA synthesis, transcription, and infected cell metabolism. Additionally, N is also thought to perform critical roles in the replication process. 419 mutations were detected in 60 countries which account for178 mutations for Europe, 121 mutations for North America, 47 mutations for South America, 46 mutations for Asia, 19 mutations for Africa and 8 mutations for Oceania (Figure 7). The D377Y (B.1) mutation that is present in the United States at a frequency of 62.98% is from North America. An aromatic ring is introduced at position 377 by the transformation of the negatively charged amino acid Aspartic Acid (D) to the aromatic polar amino acid Tyrosine (Y). By adding more contact sites, stacking and hydrogen bonding between the longer side chain of Tyrosine can potentially stabilize the structure. R203M (B.1) in Switzerland that has a frequency of 62.45%, at position 203, the amino acid Arginine (R), which is positively charged, mutates to Methionine (M), which is hydrophobic and contains sulfur. This charge change affects protein folding and interactions with nucleic acids or other proteins by disrupting electrostatic interactions and introducing a hydrophobic residue. A mutation from Aspartic Acid (D), a negatively charged amino acid, to Glycine (G), the smallest nonpolar amino acid, eliminates a charge and substantially decreases the size of the side chain. D63G (B.1) has the highest frequency in Nucleoprotein and is found in France, Europe and other parts of the world. This substitution probable dissolves the local protein structure, reducing stability (Figure 8). The mutation of Acid Aspartic to Tyrosine in position 377 with ΔΔG 0.101 kcal/mol cause stabilization of the protein structure and the mutation of Arginine to Methionine in position 203 with ΔΔG −0.252 kcal/mol and mutation of Acid Aspartic to Glycine in position 63 with ΔΔG −0.042 kcal/mol cause destabilization of the protein structure (Figure 9, Table S4). We identified twenty‐five alterations in the process of converting Threonine to Isoleucine (T>I). This process entails substituting a hydrophobic amino acid for a polar one, which lowers the possibility for hydrogen bonds to form and increases hydrophobic interactions. This process may improve structural stability. This is reversed by three mutations in the conversion of Isoleucine to Threonine (I>T), which may destabilize hydrophobic areas through the begins of a polar group. Ten variations in the amino acid conversion from Glycine to Serine (G>S) as the most sensitive amino acid conversion in various locations and nations for nucleoproteins present a hydroxyl group where Glycine supplied adaptability, which might become rigid the local structure and change its dynamics (Table S5).

### 3.12 Membrane Mutation Patterns

The M protein plays a pivotal role in the viral assembly process by engaging in protein‐protein interactions, including M‐nucleocapsid (N), M‐M, and M‐spike (S) interactions. 222 mutations were discovered in 50 countries which account for 102 mutations for Europe, 63 mutations for North America, 33 mutations for Asia, 15 mutations for Africa, 7 mutations for South America and 2 mutations for Oceania (Figure 7). With a frequency of 46.32%, the mutation A63T (XBB.1.5) in North America was found to have the highest frequency of membrane mutations in the United States. This mutation presents a polar side chain where none previously existed by changing Alanine (A), a small nonpolar amino acid, to Threonine (T), a polar amino acid with a hydroxyl group. This modification probably creates additional opportunities for hydrogen bonds, which could enhance local stability in polar conditions. The mutation of Q19E (XBB.1.5) in the membrane has been identified with frequency 46.05% in the United States from North America. This mutation provides a shift that takes over at position 19 by substituting the polar amino acid Glutamic Acid (E) for the acidic amino acid Glutamine (Q). The frequency of the I82T (B.1) membrane mutation was found to be 33.57% in the African nation of Niger. A substantial change in chemical structure happens when Isoleucine (I), a hydrophobic, nonpolar amino acid, is mutated into Threonine (T), a polar amino acid. Whereas Threonine adds a hydroxyl group that can create hydrogen bonds, Isoleucine aids in hydrophobic core contacts (Figure 8). The mutation of Alanine to Threonine in position 63 with ΔΔG 0.050 kcal/mol cause stabilization of the protein structure and the mutation of Glutamine to Acid Glutamic in position 19 with ΔΔG −0.120 kcal/mol and mutation of Isoleucine to Threonine in position 82 with ΔΔG −0.460 kcal/mol cause destabilization of the membrane structure (Figure 9, Table S4). We found eighteen changes in the membrane related to the conversion of Leucine to Phenylalanine (L>F). This conversion adds an aromatic ring into the hydrophobic core of the membrane, which may increase stiffness and stabilize interactions. There are nine mutations in the process that convert Phenylalanine to Leucine (F>L), which eliminates this aromatic interaction and increases flexibility. Eleven mutations in the process of converting Alanine to Serine (A>S) provide polarity and a hydrogen‐bonding capability where none previously appeared. This could have an effect on regional interactions and polar environment stabilization. The most vulnerable amino acids undergo two mutations in the conversion of Serine to Alanine (S>A), which alters them in different places and locations. The conversion of Serine to Alanine (S>A) reverses this, eliminating polar contacts and enhancing hydrophobic stability (Table S5).

### 3.13 ORF3a Mutation Patterns

The role of ORF3a encompasses its interactions with a number of structural proteins, namely S, M. and E. Additionally, it has been observed to induce apoptosis in vitro. 275 mutations were discovered in 48 countries which account for 125 mutations for Europe, 83 mutations for North America, 42 mutations for Asia, 11 mutations for South America, 11 mutations for Africa and 3 mutations for Oceania (Figure 7). The mutation from Threonine (T), a polar amino acid with a hydroxyl group, to Isoleucine (I), a hydrophobic, nonpolar amino acid, eliminates the possibility of bonding through hydrogen and begins a heavier hydrophobic side chain. This mutation has the highest prevalence of mutations in Orf3a, which were found in England (36.33%). The protein's capacity for participation in polar interactions is diminished by this mutation. The mutation of S26L (AY.103) in Orf3a was identified in England with a frequency of 29.94%. This alter substitutes a polar side chain with a hydrophilic one, which is capable of stabilizing the protein in hydrophobic locations, such as membrane locations. The replacement of Leucine (L), a larger hydrophobic residue, to Serine (S), a small, polar amino acid, begins the majority at position 26, reducing hydrogen bonding effectiveness. An essential change in chemical composition takes place when an amino acid, Glutamine (Q), which is polar and impartial becomes Histidine (H), which may have a positive charge depending on pH. Histidine's imidazole ring offers both hydrogen‐bonding and charge‐transfer capacities, which makes this genetic change probably to affect the local electrostatic environment. This mutation of Q57H (BQ.1.1) in Orf3a was observed in the United States from North America with a frequency of 3.68% (Figure 8). The mutation of Threonine to Isoleucine in position 223 with ΔΔG 0.061 kcal/mol and Glutamine to Histidine in position 57 with ΔΔG 0.774 kcal/mol cause stabilization of the ORF3a protein structure (Figure 9, Table S4). The conversion of Leucine to Phenylalanine (L>F) includes the replacement of a hydrophilic aliphatic side chain with an aromatic one, which brings stiffness and possible stacking interactions. We identified twenty‐three mutations in this process. Six mutations in the conversion of Phenylalanine to Leucine (F>L), which is the most sensitive amino acid conversion for ORF3a in various places and nations, have the opposite effect of improving flexibility by eliminating the aromatic ring. According to the local context, these changes can affect the packing and stability of proteins, especially in hydrophobic or membrane‐associated locations (Table S5).

### 3.14 ORF7a Mutation Patterns

The induction of apoptosis, inhibition of cellular protein synthesis, and arrest of the cell cycle at the GO/G1 phase are all attributed to the action of ORF7a. 121 mutations were recognized in 32 countries which account for 49 mutations for Europe, 39 mutations for North America, 21 mutations for Asia, 6 mutations for Africa, 4 mutations for South America and 2 mutations for Oceania (Figure 7). The most frequent mutations of ORF7a were found in the conversion of T120I (19 A), which happened with a frequency of 28.50% in France. The mutation changes a side chain that can form hydrogen bonds with a larger, hydrophobic one, replacing a polar amino acid called Threonine (T) to a hydrophobic, nonpolar amino acid called Isoleucine (I). This substitution is anticipated to disrupt local hydrogen bonding interactions by substituting hydrophobic wrapping for the side chain. The discovery of the mutation of V82A (AY.103) in ORF7a was made with a frequency of 28.27% in France, Europe. The mutation reduces side‐chain large quantities at position 82 by changing the hydrophobicity and size of Valine (V), a medium‐sized hydrophobic amino acid, to Alanine (A), a smaller, nonpolar amino acid. A mutation of P45L (XBB.1.5.12) in ORF7a was found to occur with a frequency of 1.11% in Saudi Arabia from Asia. This mutation begins more conformational adaptability by replacing the rigid amino acid Proline (P) with the flexible hydrophobic amino acid Leucine (L). Proline usually causes bends or turns in protein structures, so replacing it with Leucine could relieve these limitations and alter the process of secondary structure formation, such as helix stabilizing (Figure 8). The mutation of Valine to Alanine in position 82 with ΔΔG −0.117 kcal/mol and mutation of Proline to Leucine in position 45 with ΔΔG −0.155 kcal/mol cause destabilization of the ORF7a protein structure (Figure 9, Table S4). Ten mutations were found in the conversion of Phenylalanine to Leucine (F>L), and four mutations were found in the conversion of Leucine to Phenylalanine (L>F). These mutations include the exchange of hydrophobic residues between Phenylalanine and Leucine (F>L) and Leucine and Phenylalanine (L>F). However, the aromatic ring of Phenylalanine provides stiffness and may facilitate arranging connections. Structural stiffness is introduced when the aromatic ring is removed (F>L) and reduced when it is removed in reverse (L>F). Nine mutations happen in the conversion of Threonine to Isoleucine (T>I), with the conversion of amino acids being most sensitive in various countries and geographical areas for ORF7a. T>I presents hydrophobic side chains where polar side chains before existed, affecting the potential for hydrogen bonds and probably changing the stability of the protein or its interaction with other proteins (Table S5).

### 3.15 ORF7b Mutation Patterns

43 mutations were discovered in 18 countries including 22 mutations for Europe, 12 mutations for North America, 8 mutations for Asia and 1 mutation for South America (Figure 7). The conversion of T40I (AY.103), Threonine, a polar amino acid with a hydroxyl group, to Isoleucine (I), a hydrophobic, nonpolar amino acid, which substitutes a polar side chain with a hydrophobic one, is the most common mutation in ORF7b, occurring at a frequency of 27.56% in Guangdong Province, China. This alteration may affect the stability and folding of the protein by displacing hydrogen bonding interactions with hydrophobic interactions. L11F (BA.4) from Asia that occurs in India with a frequency of 1.30%. Rigidity is added at position 11 by the transformation of the hydrophobic amino acid Leucine (L) to the hydrophobic amino acid Phenylalanine (F), which has an aromatic ring. The stacking interactions that the aromatic arrangement of Phenylalanine might participate in might impact how hydrophobic residues are packed. H42Y (BQ.1.1) from Europe that is discovered in France at a frequency of 0.15%. The side chain characteristics at position 42 are significantly altered when Tyrosine (Y), a polar aromatic amino acid, replaces Histidine (H), an amino acid that can be positively charged depending on pH. The aromatic ring of Tyrosine makes it bigger and stiffer, which may facilitate stacking connections. The structural dynamics of ORF7b may be impacted by this modification, which could also influence the hydrogen bonding capacity and local electrostatic environment (Figure 8). The mutation of Leucine to Phenylalanine in position 11 with ΔΔG −0.378 kcal/mol cause destabilization of ORF7b protein structure (Figure 9, Supplementary Table S4). Four mutations were identified in the process of converting Leucine to Phenylalanine (L>F). This process includes the substitution of a larger aromatic ring for a hydrophobic aliphatic side chain, which increases stiffness and may change the hydrophobic core packing. Four mutations that convert Leucine to Serine (L>S) introduce a polar side chain instead of a hydrophobic one, which may disrupt the hydrophobic regions and change the stability of the protein. Two mutations in ORF7b that cause Serine to Leucine (S>L) conversion in various positions and countries are the most important amino acid alteration. This conversion enhances wrapping in hydrophobic regions but decreases adaptation and hydrogen‐bonding capacity (Table S5).

### 3.16 ORF8 Mutation Patterns

ORF8 has been determined to augment replication in certain investigations and demonstrates association with other structural proteins. 121 mutations were detected in 42 countries which account for 60 mutations for Europe, 30 mutations for North America, 18 mutations for Asia, 6 mutations for South America, 6 mutations for Africa and 1 mutation for Oceania (Figure 7). With a frequency of 2.05% in Switzerland, the conversion of S24L (BA.5.3.1), Serine to Leucine (L), a larger hydrophobic amino acid, was found to have the highest mutation frequency in ORF8. This mutation removes the hydrogen bonding an opportunity and presents a hydrophobic side chain at position 24, replacing Serine (S), a small polar amino acid. This alteration most likely improves ORF8's hydrophobic contacts, which supports the stability of the protein core. The mutation E92K (BQ.1.10.1), which occurs with a frequency of 1.48% in England, begins an opposite charge at position 92 by switching from the negatively charged amino acid Glutamic Acid (E) to the positively charged amino acid Lysine (K). This change in charge probably interferes with the electrostatic interactions that are already in place and replaces them with new ionic bonds, which may lead to protein destabilization. Russia has a frequency of 0.73% for the T11I (XBB.1.5) mutation that originated in Europe. The substitution of Isoleucine (I), a nonpolar amino acid, for Threonine (T), a polar amino acid with a hydroxyl group, results in the elimination of hydrogen bonding possible and the begins of a larger hydrophobic residue. This modification decreases flexibility and modifies regional hydrophobic wrapping, potentially improving the integrity of the structure (Figure 8). The mutation of Serine to Leucine in position 24 with ΔΔG 1.349 kcal/mol cause stabilization of ORF8 protein structure and mutation of Acid Glutamic to Lysine in position 92 with ΔΔG −0.142 kcal/mol cause destabilization (Figure 9, Table S4). In ORF8, we found three mutations in the conversion of Histidine to Tyrosine (H>Y), which enhances stacking interactions by introducing an aromatic ring where Histidine's imidazole group previously made it possible for proton exchange. Three mutations in the Tyrosine to Histidine (Y>H) conversion provide adaptability due to the varied protonation of Histidine. One mutation in the conversion of Lysine to Acid Glutamic (K>E) and three mutations in the conversion of Acid Glutamic to Lysine (E>K) must be especially concerned about the conversion of amino acids in various locations and declares that electrostatic interactions are drastically changed when Glutamic Acid (E) and Lysine (K) are substituted. This replace of negatively and positively charged residues might stabilize or destabilize electrostatic connections, depending on the surrounding conditions (Table S5).

## 4 Discussion

This study provides a comprehensive analysis of immune cell dynamics and viral gene expression in SARS‐CoV‐2 infection, revealing distinct cellular and molecular profiles across healthy, mild and severe conditions. The predominance of Neutrophils and Monocyte:CD14+ in both healthy‐mild and healthy‐severe comparisons, as observed through single‐cell RNA sequencing and spatial transcriptomics, underscores their critical role in driving inflammation, particularly in central and upper‐left lung tissue regions. Neutrophils and Monocyte:CD14+ are consistently enriched, especially in inflammatory lung areas, suggesting that they play a crucial role in coordinating inflammatory responses during SARS‐CoV‐2 infection [46]. These cells may be candidates for treatments aimed at reducing chronic inflammation in COVID‐19 and related infectious disorders due to their continued existence across disease severities and indicating to a widespread mechanism of immune activation [46]. The increased proportions of T‐cells (CD4+ central memory, naive) and NK cells in mild and severe samples suggest an enhanced adaptive immune response as infection progresses, while the reduced abundance of Monocyte:CD14+ and Platelets in severe cases may reflect immune exhaustion or tissue damage [47]. Trajectory analysis further highlights the progression of immune cells, with Neutrophils and Monocyte:CD14+ initiating responses, followed by T‐cells (CD4+ naive, gamma‐delta) and NK cells dominating later stages, indicating a shift from innate to adaptive immunity. To better manage inflammation and virus elimination, this development highlights to an organized immune response that changes during time and offers insight into possible therapeutic opportunities for targeting particular cell types [48]. Cell‐cell interaction networks reveal strong signaling via MHC‐I, MIF, and CD99 pathways, with T cell:CD4+ effector memory and NK cells playing key roles in immune coordination, particularly in mild cases. MHC‐I signaling facilitates antigen presentation to cytotoxic T cells, enabling the immune system to recognize and eliminate virus‐infected cells, which is essential for antiviral defense [49]. Immunological cell adhesion and migration across endothelial barriers are influenced by CD99 signaling, and elevated activity in severe infections can outcome in insufficient immunological infiltration and tissue inflammation [50]. MIF signaling maintains the production of cytokines and macrophage activation, which increases pro‐inflammatory responses and may increase immune‐driven tissue damage in severe infections [51]. Spatial transcriptomics analysis of viral gene expression in samples V10B13‐401 and V10L13‐003 shows heterogeneous patterns, with genes N and ORF1ab exhibiting high expression in active replication zones, while genes like ORF10 display negligible activity, reflecting their limited role in pathogenesis. Spatial analysis revealed that areas with the highest infiltration of Neutrophils, Monocyte:CD14 + , and T cell:CD4+ central memory also exhibited elevated expression of several key SARS‐CoV‐2 genes, including Spike, Envelope, Nucleoprotein, Membrane, ORF1ab, and ORF8. This spatial overlap suggests that immune cells are recruited to regions of active viral replication, highlighting a localized immune response that may contribute to tissue pathology or viral clearance. These genes are located in regions with high levels of inflammation and immune cell infiltration, and they are crucial for viral RNA assembly and replication, respectively [52]. Understanding the tissue‐specific expression of these viral genes not only sheds light on the mechanisms of pathogenesis but also highlights potential targets for localized antiviral therapies, particularly those aimed at inhibiting replication‐related proteins such as N and ORF1ab [53]. These findings collectively highlight the complex interplay between immune cell dynamics and viral activity, providing insights into regional immune responses and potential therapeutic targets for mitigating SARS‐CoV‐2‐induced pathology. During the early stages of the pandemic, the virus swiftly moved throughout the world and continued to spread globally. There was a more diversified mix of strains among the samples submitted to GISAID at that time [54, 55, 56]. Type G strains became more prominent in nations with higher resistance, resulting in a strain shift [57]. Based on previous reports, the G strains may be a simpler target for vaccination because they contain larger quantities of surface proteins that target antibodies that bind to vaccines. Spike protein enables virus attachment to the human cell surface ACE2 receptor, allowing for viral entrance during infection [58, 59, 60]. Spike protein divided into two components are called S1 and S2. The S1 unit has a receptor‐binding domain (RBD) that may directly connect to the ACE2 receptor. It is the primary target of SARS‐CoV‐2 neutralizing antibodies (Ab). As a result, S1 is a hotspot for mutations with significant clinical significance in virulence, transmissibility, and host immune evasion [5, 61, 62, 63]. The Alpha version features a N501Y mutation, which means that N asparagine has been replaced with Y tyrosine at the 501 residues [64]. Other than N501Y, Beta and Gamma versions feature additional substitutions [64]. The AA change in D614G has been documented as a result of Acid Aspartic to Glutamic Acid mutation in position 614 in the Wuhan reference strain. In early March 2020, this mutation was the first known alteration in the Spike analysis. The D614G mutation is frequently seen in conjunction with two additional mutations; C to T mutation at position 241 in the ‘5 UTR compared to the Wuhan reference sequence (silent C to T mutation at viral RdRp site) (P323L RdRp) [65]. G476S mutation were discovered in the United States samples initially, whereas the V367F mutant was found in Switzerland. The mutations V367F and D364Y have been shown to improve the stability of the Spike protein structure and promote effective binding to the ACE2 receptor [14]. Orf8 is a appropriate location for viral coronavirus alterations, according to research by Young E Barnaby et al [66]. Deletion in this area appears to result in a milder illness with less systemic pro inflammatory cytokine production and a more effective immune response to SARS‐CoV‐2 [66]. Further research into this mutation might help to further therapy and vaccine development [66]. NSP1 is a critical protein that inhibits type I interferon activation in the host while promoting viral replication [64, 67]. Regarding NSP12, Goldswain H and et al [68] revealed that mutation of Proline to Leucine in position 323 can impact various aspects of the virus, including its transmissibility, virulence, or response to treatments and vaccines [69], which are consistent with our research results. Lin X and et al [70], reported that in the NSP4, mutation of Threonine to Isoleucine in position 492 associates with attenuated pathogenicity of SARS‐CoV‐2 variants of concern including Delta and Omicron and T492I mutation increases SARS‐CoV‐2 transmissibility and adaptability, which this study is consistent with our findings. Yuan‐Qin Min et al, revealed that in the NSP1 protein, the conversion of Aspartic Acid to Glutamic Acid occurred in position 75 in China. However, in our study, the conversion of Glutamic Acid to Aspartic Acid occurred in position 87 in England, the present research is not consistent with our analyses [71]. Orf8 is an immune‐evasive protein that reduces the expression of major histocompatibility complex class I (MHC‐I) in host cells [66]. Recently, it was discovered that the Alpha variation found from a single immunocompromised person had a premature stop codon in position 27 of Orf8 [13]. Spike protein mutation D614G has influenced the SARS‐CoV‐2 transmissibility frequency due to increased affinity for olfactory epithelium, it has been shown to have higher transmissibility in animal models [72, 73]. The Alpha variation has been reported to increase hospitalizations and death, which may be attributable to their resistance to neutralizing antibodies owing to RBD mutations [74]. Yuan and colleagues discovered that the CR3022 neutralizing antibody derived from a SARS patient binds to the RBD region of the spike in the SARS‐CoV‐2 virus by producing the crystal structure of this complex [75]. This crystal structure enables researchers to comprehend how the SARS‐CoV‐2 virus is discovered. Identifying and obtaining these epitopes enables the designing of vaccines against the SARS‐CoV‐2 virus and the detection of antibodies to additional coronaviruses in the future [75]. Wang and colleagues have identified 13 mutation sites in the SARS‐CoV‐2 Orf1ab, Orf3a, Orf8, and N regions, with 28144 in Orf8 and 8782 Orf1a showing a mutation frequency of 30.53% and 29.47%, respectively [76]. The SARS‐CoV‐2 RdRp (also known as NSP12) is a crucial component of the replication‐transcription machinery [77]. We found ten mutations for Orf7a protein which, according to a recent study, showed that ectodomain Orf7a binds to monocytes in the human peripheral blood, reducing its ability to deliver antigens, inducing dramatic expression of pro‐inflammatory cytokines and since the lungs are the site of SARS‐CoV‐2 proliferation causing the accumulation of monocytes in the lungs [78]. Also, the Orf8 signaling pathway promotes pro‐inflammatory by activation of IL‐17, which contributes to cytokine storm in SARS‐CoV‐2 infection [79], includes apoptosis [80] and antagonizes the IFN signaling pathway [81]. It has been shown that Orf9b could cause the inflammasome's activation and significant antibody responses have been associated with Orf9b. On the other hand, Orf9c expression impaired interferon signaling, antigen processing and presentation, complement signaling, and induced IL‐6 signaling [82]. Our study reported that the T60A mutation, which is correlated to Orf9b, is the more frequent mutation in France, however, Gopika et al, also found this mutation in Thailand [83]. Gupta S et al, concluded that in the Orf3a protein, Serine to Leucine conversion occurred in position 171 in India, however, we observed this AA conversion occurrence in the United States with a frequency of 29% [84]. Michael D Sacco et al, revealed that mutations such as: conversion of Proline to Histidine in position 132, conversion of Leucine to Phenylalanine in position 89 and conversion of Lysine to Arginine in position 90 for the NSP5 protein in the omicron strain was consistent with our studies [85]. The potential of SARS‐CoV‐2 to avoid immune detection through alterations in both structural and nonstructural proteins, along with its continuing mutation across many locations, highlight the necessity for continual research and adaption of public health methods to effectively combat emerging variations.

## 5 Conclusion

This study highlights the critical role of integrating single‐cell and spatial transcriptomics to deepen our understanding of SARS‐CoV‐2 dynamics, paving the way for improved therapies and early variant detection to manage the global pandemic. We observed distinct immune shifts, with Neutrophils and Monocyte:CD14+ predominant in mild and severe cases, alongside elevated T‐cell (CD4+ central memory, naive) and NK cell proportions, reflecting a robust adaptive immune response, while spatial analysis revealed high N and ORF1ab gene expression in viral replication hotspots. The lack of detailed metadata from GISAID limited our ability to stratify mutations by age or sex, a challenge future studies should address by incorporating comprehensive clinical data. Our global mutation analysis of 15,669,529 samples underscores the need for region‐specific mutation profiles, considering factors like age and gender, to inform tailored vaccine and primer development. Furthermore, cell‐cell interaction networks identified T cell:CD4+ effector memory and NK cells as key immune coordinators, suggesting new therapeutic avenues. By combining geographical mutation tracking with molecular insights, this study lays a foundation for strategic interventions to curb transmissibility, morbidity, and mortality, enhancing COVID‐19 diagnostics, vaccines, and treatments.

## 6 Limitations

This study is limited by the small number of single‐cell (*n* = 12) and spatial transcriptomic (*n* = 5) samples, which may affect statistical power and generalizability. Data set selection was based on data quality and the rare availability of integrated human and viral gene expression. While these constraints limit interindividual comparisons, the analysis still provides valuable insights. Future studies with larger cohorts are needed to validate and expand upon our findings.

## Author Contributions

Conceptualization: Mohammadamin Mahmanzar, Karim Rahimian, Saleha Bayat, Seyed Taleb Hosseini and Youping Deng. Methodology: Mohammadamin Mahmanzar, Karim Rahimian, and Seyed Taleb Hosseini. Software: Mohammadamin Mahmanzar, Karim Rahimian, and Seyed Taleb Hosseini. Validation: Mohammadamin Mahmanzar, Karim Rahimian and Donna Lee Kuehu. Formal analysis: Mohammadamin Mahmanzar, Karim Rahimian, and Seyed Taleb Hosseini. Investigation: Mohammadamin Mahmanzar, Seyed Taleb Hosseini, Mahsa Mollapour Sisakht and Amin Farhadi. Resources: Karim Rahimian. Data curation: Mohammadamin Mahmanzar, Karim Rahimian, Seyed Taleb Hosseini, and Donna Lee Kuehu. Writing original draft preparation: Seyed Taleb Hosseini and Amir Gholamzad. Writing review and editing, Mohammadamin Mahmanzar and Donna Lee Kuehu. Visualization: Saleha Bayat and Seyed Taleb Hosseini. Supervision: Youping Deng. Project administration: Mohammadamin Mahmanzar and Youping Deng.

## Conflicts of Interest

The authors declare no conflicts of interest.

## Supporting information

Supplementary Text.

**Supplementary Figure S1:** Spatial autocorrelation analysis of viral gene expression in lung tissue samples from COVID‐19 patients.

**Supplementary Figure S2A:** The top 10 mutations of genes (NSP1‐NSP9) such as: mutation position, mutation frequency, country name, mutation date and continent.

**Supplementary Figure S2B:** The top 10 mutations of genes (NSP10‐NSP16, Spike and ORF3a) such as: mutation position, mutation frequency, country name, mutation date and continent.

**Supplementary Figure S3:** Results of the DynaMut server to investigate the effect of mutations on the stability or instability of the SARS‐CoV‐2 proteins including NSP1 to NSP14. V10B13‐401.

Supplementary Fiugre's Caption.

**Supplementary Table S1:** Percentage and Number of Counts for Cell Types. **Supplementary Table S2:** Number of samples by continent for genes. **Supplementary Table S4:** Predictions Outcomes from Dynamut Server (ΔΔG‐ = destabilizing and ΔΔG+ =stabilizing). **Supplementary Table S5:** Most susceptible amino acids conversion in different positions and countries with different rates.

Supplementary Table S3.

## Data Availability

The data that supports the findings of this study are available in the Supporting material of this article. Publicly available datasets were analyzed in this study. These data can be found at GSE216020 from Gene Expression Omnibus (GEO, https://www.ncbi.nlm.nih.gov/geo/) and https://doi.org/10.5281/zenodo.8039011 for spatial transcriptomics data. All other data supporting the findings of this study are available within the article and the supporting information or from the corresponding author upon reasonable request.
