# Supplementary material for: Spatial Transcriptomics and Single Cell‐RNASeq Reveals Cellular Heterogeneity of SARS‐CoV‐2 in Lung Tissues and Global Mutational Patterns in COVID‐19 Patients
Source: J Med Virol. 2025 Sep 5;97(9):e70586. doi: 10.1002/jmv.70586 (PMC12412077; doi:10.1002/jmv.70586)
Supplement: Supplementary file 1 — Supplementary Text. [file JMV-97-e70586-s002.pdf]

Supplementary Text

NSP1 Mutation Track

The inhibition of host translation, the induction of host immune response evasion, and the promotion of optimal viral gene expression in infected cells are all attributed to the NSP1 protein. We found 180 mutations for 20 countries including 143 mutations for North America, 23 mutations for Europe, 7 mutations for Asia, 3 mutations for South America, 3 mutations for Africa and 1 mutation for Oceania (**Figure 7**). S135R (19A), which has a 30.81% frequency in France, is the most common mutation in NSP1. A big side chain with numerous hydrogen-bonding and electrostatic interaction capabilities is introduced when Serine (S), a polar amino acid with hydroxyl activity, is mutated to Arginine (R), a positively charged basic amino acid. This modification most likely modifies the protein's surface charge and impairs local structural flexibility, perhaps changing interaction locations (**Supplementary Figure S2A**). K47R (19A) in Brazil with a frequency of 2.56% A moderate alteration is the shift from the positively charged amino acid Lysine (K) to the similarly positively charged amino acid Arginine (R). Arginine and Lysine are both basic amino acids, but arginine has a bigger side chain that has a greater potential for hydrogen bonds, which could somewhat change how proteins interact (**Supplementary Figure S2A**). E87D (XBB.1) in England has a frequency of 0.64%. Side-chain length is reduced by the mutation of Aspartic Acid (D), another negatively charged amino acid, from Glutamic Acid (E), a negatively charged acidic amino acid. Since Aspartic Acid's chain is shorter than Glutamic Acid's, electrostatic interactions and hydrogen bonding in this area may be impacted (**Supplementary Figure S2A**). Our results show that the mutation of Serine to Arginine in position 135 with energy $\Delta\Delta G$ -0.125 kcal/mol, mutation of Lysine to Arginine in position 47 with energy $\Delta\Delta G$ -0.394 kcal/mol and mutation of Acid Glutamic to Acid Aspartic in position 87 with energy $\Delta\Delta G$ -0.518 kcal/mol can result in destabilizing the structure of NSP1 (**Supplementary Figure S3**, **Supplementary Table S4**). We discovered five mutations in NSP1 that affect the conversion of Leucine to Phenylalanine (L->F). This conversion results in the begins of an aromatic ring, which stiffens the protein and may improve interactions between hydrophobic cores. One mutation in the process of converting Phenylalanine to Leucine (F->L) eliminates the aromatic component, which may increase flexibility. Four mutations occur in the conversion of Alanine to Valine (A->V), which strengthens hydrophobic bonds by introducing a larger, stronger hydrophobic residue. The most sensitive amino acid conversions are Valine to Isoleucine (V->I), that includes four changes in numerous locations and nations worldwide. While this conversion entails a slight change in side-chain bulk, it most likely maintains overall stability while slightly altering hydrophobic packing (**Supplementary Table S5**).

NSP2 Mutation Track

The NSP2 protein exhibits interaction with the host protein complexes PHB1 and PHB2, both of which play a crucial role in the process of mitochondrial biogenesis. 637 mutations were detected for NSP2 in 46 countries which account for 483 mutations for North America, 108 mutations for Europe, 28 mutations for Asia, 11 mutations for Africa, 5 mutations for South America and 2 mutations for Oceania (**Figure 7**). The Q376K (BQ.1.1) mutations are European and have a frequency of 4.04% in Ireland. A major charge shift occurs at position 376 when the positively charged basic amino acid Lysine (K) replaces the polar amino acid Glutamine (Q). This mutation improves electrostatic interactions because Lysine is positively charged (**Supplementary Figure S2A**). T85I (BA.4.6) from North America with a frequency of 2.92% in the US A possible hydrogen bond donor is eliminated and replaced with a large hydrophobic side chain when Threonine (T), a polar amino acid with a hydroxyl group, is substituted for Isoleucine (I), a hydrophobic amino acid. This modification modifies local hydrophobic packing (**Supplementary Figure S2A**). The frequency of K81N (BQ.1.1.69), which was among the highest in NSP2, was 1.60% in the Democratic Republic of the Congo. At position 81, a polar group is introduced and the positively charged amino acid Lysine (K) is mutated to the neutral polar amino acid Asparagine (N). This alteration interferes with electrostatic interactions (**Supplementary Figure S2A**). The mutation of Glutamine to Lysine in position 376 with ΔΔG 0.905 kcal/mol cause stabilization of the protein structure, Threonine to Isoleucine in position 85 with ΔΔG -0.264 kcal/mol and mutation of Lysine to Asparagine in position 81 with ΔΔG -0.155 kcal/mol cause destabilization of the NSP2 structure (**Supplementary Figure S3**, **Supplementary Table S4**). For NSP2, the conversion of Threonine to Isoleucine (T->I) contains twenty mutations that we were able to identify. T->I reduce the potential for hydrogen bonds to form and increases hydrophobic interactions, which may result in structural stabilization or modifications to protein-protein interactions. Eight mutations in the Isoleucine to Threonine (I->T) conversion that on the other hand, the begins of a polar group into a hydrophobic area during the Isoleucine to Threonine (I->T) transition may result in instability. The conversion of Alanine to Valine (A->V) involves sixteen mutations that introduce larger side chains while retaining hydrophobic contacts, maybe improving stability marginally and There are four mutations in the most vulnerable amino acid conversion, Valine to Alanine (V->A), which decreases hydrophobic packing in multiple locations (**Supplementary Table S5**).

NSP3 Mutation Track

The NSP3 molecule facilitates the release of NSP1 and NSP2 from the polyprotein. Additionally, it interacts with other viral non-structural proteins as well as RNA molecules to create a replication/transcription complex. Lastly, NSP3 plays a crucial role in removing tags from old proteins that are marked for degradation. A total of 1945 mutations were discovered in 62 countries,1579 mutations for North America, 269 mutations for Europe, 61 mutations for Asia, 18 mutations for Africa, 10 mutations for South America and 8 mutations for Oceania (**Figure 7**). In NSP3, the top three conserved mutations were detected in different countries. These mutations are rated from 40% to 33%. The mutation at position 489 (XBB.1.5) in Guangdong, Asia, is characterized by a frequency of 40.29%. It results in a change from Glycine (G), a small and flexible amino acid, to Serine (S), a slightly larger amino acid with a hydroxyl group. This substitution introduces the possibility of hydrogen bonding at this position (**Supplementary Figure S2A**). T24I (XBB.1.5), which occurs with a frequency of 40.23% in France, is a mutation that results in the begins of a longer, hydrophobic side chain and the removal of a hydrogen-bonding capability from Threonine (T), a polar amino acid with a hydroxyl group. This replacement makes hydrophobic interactions stronger (**Supplementary Figure S2A**). P1228L (AY.103) from Europe, with a frequency of 33.59% in England, and at position 1228, inflexibility is decreased by a mutation from the bigger a hydrophobic amino acid Leucine (L) to the rigid amino acid Proline (P), which is known to introduce kinks in the polypeptide chain. This substitution increases flexibility in the structure of protein, which could influence the local convertible dynamics or interactions with other proteins or RNA (**Supplementary Figure S2A**). The mutation of Threonine to Isoleucine in position 24 with ΔΔG 0.131 kcal/mol cause the stabilization of the NSP3 structure (**Supplementary Figure S3, Supplementary Table S4**). In NSP3, we found sixty- five alteration in the conversion of Threonine to Isoleucine (T->I) are responsible for the begins of a hydrophobic residue in place of a polar one, which prevents possible hydrogen bonding and increases the stability of the hydrophobic core, perhaps leading to structural stabilization. The most responsive amino acid alteration has been accomplished by eighteen mutations in the conversion of Isoleucine to Threonine (I->T). Isoleucine to Threonine (I->T) introduces a polar group into the hydrophilic region, potentially causing instability the structure by affecting hydrophobic transportation (**Supplementary Table S5**).

NSP4 Mutation Track

The proteins NSP4 are anticipated to possess the ability to initiate and firmly attach viral replication complexes onto double-membrane vesicles situated within the cytoplasm. 500 mutations were recognized in 27 countries including 420 mutations for North America, 49 mutations for Europe, 20 mutations for Asia, 6 mutations for Africa, 3 mutations for South America and 2 mutations for Oceania (**Figure 7**). The highest frequency of mutations in Europe and Asia was found in Wuhan, India, and Switzerland (mutations T492I (XBB.1.5), with a frequency of 79.45% in Switzerland. This mutation alters the polarity at this site by changing Threonine (T), a polar, uncharged amino acid, to Isoleucine (I), a non-polar, hydrophobic amino acid. The protein's propensity to interact with membrane lipids or other hydrophobic areas may be enhanced by this change to a more hydrophobic residue, which could stabilize the protein's attachment to double-membrane vesicles and influence the assembly of the viral replication complex (**Supplementary Figure S2A**). L264F (XBB.1.5) in India at a frequency of 35.81%. The arrival of Phenylalanine (F), another hydrophobic residue with a larger aromatic ring, in place of the non-polar, hydrophobic amino acid Leucine (L), results in a major structural alteration because of the Phenylalanine side chain's increased size and rigidity. This mutation may have an effect on how the protein packs or interacts with neighboring residues, which could have an effect on NSP4's local folding or stability (**Supplementary Figure S2A**). T327I (XBB.1.5) in Wuhan at a frequency of 35.78% This substitution of Isoleucine (I) for Threonine (T) results in the begins of a hydrophobic residue in place of a polar one, much like the T492I mutation. It is possible that this alteration stabilizes membrane connections or interactions with other nonpolar proteins by decreasing the protein's capacity to generate hydrogen bonds in this area and increasing its interaction with hydrophobic environments (**Supplementary Figure S2A**). For NSP4, there are nine mutations in the conversion of Leucine to Phenylalanine (L->F), seven mutations in the conversion of Phenylalanine to Leucine, while both Leucine and Phenylalanine are hydrophilic, the aromatic structure of Phenylalanine adds more rigidity and an opportunity stacking interactions that could change protein shape. The most vulnerable amino acid conversions are eight mutations in the conversion of Serine to Phenylalanine (S->F) and four mutations in the conversion of Phenylalanine to Serine (F->S) at various positions and geographical areas. additionally, Similarly, Serine to Phenylalanine substitutions results in the beginning of a hydrophobic residue in place of a polar one, which may decrease the capacity for hydrogen bonding and have an impact on interactions between proteins or lipids, especially in areas of the protein that are connected with membranes (**Supplementary Table S5**).

NSP5 Mutation Track

The mature enzyme is produced by the initial automatic cleavage of NSP5 from polyproteins. Subsequently, this mature enzyme cleaves downstream NSPs at 11 sites, thereby releasing nsp4-nsp16. 306 mutations were found in 15 countries which account for 268 mutations for North America, 23 mutations for Europe, 11 mutations for South America, 2 mutations for Africa, 1 mutation for Asia and 1 mutation for Oceania (**Figure 7**). A frequency of 51.26% for P132H to NSP5 mutations were the highest in the United States. At this location, flexibility occurs via the change of Histidine (H), a polar, positively charged amino acid, to Proline (P), a rigid, non-polar amino acid. Histidine can participate in acid-base interactions and form hydrogen bonds, but Proline is unique in that it causes a kink in the protein backbone because of its cyclic structure. It is possible that this alteration will stabilize the overall protein structure by reducing the stiffness of the local structure and increasing flexibility and interaction potential (**Supplementary Figure S2A**). In Switzerland, K90R has a frequency of 1.37%. It takes two basics, positively charged amino acids to convert Lysine (K) to Arginine (R). Arginine can, however, form stronger hydrogen bonds and salt bridges because to its longer and more complex side chain. This alteration is comparatively cautious in terms of charge, but the longer side chain of Arginine could impact the structure of proteins or its interactions with other compounds (**Supplementary Figure S2A**). L89F from North America that has a frequency of 1.13% in the US. The regional structure is drastically changed by the mutation of Leucine (L), a small a hydrophobic residue, to Phenylalanine (F), a bigger hydrophobic residue with a ring of aromatic compounds. The inclusion of Phenylalanine's large aromatic rings likely influences the packing arrangement of the protein, which could contribute to a destabilizing of the natural structure (**Supplementary Figure S2A**). The mutation of Proline to Histidine in position 132 with ΔΔG 1.698 kcal/mol cause stabilization of the protein structure, mutation of Lysine to Arginine in position 90 with ΔΔG -0.033 kcal/mol and mutation Leucine to Phenylalanine in position 89 with ΔΔG -0.852 kcal/mol cause destabilization of the NSP5 structure (**Supplementary Figure S3**, **Supplementary Table S4**). There are seven mutations in NSP5 that cause the conversion of Alanine to Valine (A->V). Valine is heavier than Alanine and can cause alterations in local folding and tighter packing. Three mutations occur in the conversion of Asparagine to Threonine (N->T). Asparagine (N) is a polar, uncharged residue, whereas Threonine (T) is polar as well, but it has a hydroxyl group that makes it able to form hydrogen bonds. These hydrogen bonds have the ability to drastically change the local configuration of proteins as well as how they connect to other molecules that are polar or water. One alteration in the process of converting Threonine to Asparagine (T->N) was found to be the most delicate because it eliminates the hydroxyl group, which may lower the protein's ability to form hydrogen bonds (**Supplementary Table S5**).

NSP6 Mutation Track

The NSP6 proteins are anticipated to possess the ability to initiate and firmly attach viral replication complexes onto double-membrane vesicles situated within the cytoplasm. 290 mutations were discovered in 30 countries which account for 205 mutations for Asia, 34 mutations for North America, 31 mutations for Europe, 10 mutations for South America, 9 mutations for Africa and 1 mutation for Oceania (**Figure 7**). Among the top three NSP6 mutations, T77A (AY.103) has the highest prevalence (70.37%) Guangdong Province from China. Polarity is eliminated from the location via the transformation of Alanine (A), a little, non-polar hydrophobic amino acid, for Threonine (T), a polar, uncharged amino acid. In a previously polar environment, this conversion decreases the capacity to form hydrogen bonds and introduces a more hydrophobic residue. Since Alanine promotes a more hydrophobic contact and Threonine can interact with water or other polar molecules, this could change how NSP6 interacts with other proteins or membranes. It may also change how NSP6 folds proteins or associates with membranes (**Supplementary Figure S2A**). V149A (B.1) in Guangdong Province, China, with a frequency of 6.88% and the bulkiness of the side chain is reduced by replacing the hydrophobic amino acid Valine (V) with the smaller, hydrophobic residue Alanine (A). This mutation modifies the local conformation or permits tighter packing by marginally reducing the steric barrier in the protein structure (**Supplementary Figure S2A**). The alteration, meanwhile, is still contained in a hydrophobic environment, which might have little effect on the protein's total hydrophobic interactions and the mutation T181I (19A), which is found in North America and has a frequency of 5.71% in the United States, replaces a polar residue with a considerably bigger hydrophobic one when it switches from Threonine (T) to Isoleucine (I). The protein may interact with surrounding molecules or membranes quite differently as a result of this substantial change from a polar to a hydrophobic environment. NSP6's interactions with other elements of the viral replication complex and its ability to bind to double-membrane vesicles may be impacted by its diminished hydrogen bonding capacity (**Supplementary Figure S2A**). Ten variations in the conversion of Leucine to Phenylalanine (L->F) and three mutations in conversion of Phenylalanine to Leucine (F->L) were found. Although both Phenylalanine and Leucine are hydrophobic, the aromatic group of Phenylalanine increases weight and stiffness, which may alter protein folding and interactions between hydrophobic molecules. The strongest amino acid conversions occur in eight instances with Alanine to Valine (A->V) and one instances of Valine to Alanine (V->A). These changes involve small-to moderately big hydrophobic residues, which may affect folding of proteins by modifying the degree to which various components of the protein may be packed collectively. These changes are probably going to have an impact on membrane contacts that are essential to NSP6 activity, particularly in polar areas (**Supplementary Table S5**).

NSP7 Mutation Track

The dimerization of SARS-CoV nsp7 and its interaction with various other proteins, namely nsp5, nsp8, nsp9, and nsp13, have been observed. 83 mutations were detected in 9 countries such as 69 mutations in North America, 11 mutations in Europe, 1 mutation for Asia, 1 mutation for South America and 1 mutation for Africa (**Figure 7**). The most common NSP7 mutations are L71F (BQ.1.1), which has a frequency of 0.18% in England and is found in Europe, and A greater side chain is added when Leucine (L), an extremely small, hydrophobic amino acid, is mutated to Phenylalanine (F), a bigger, hydrophobic residue containing an aromatic ring. This increase in size and stiffness probably strengthens the protein's hydrophobic interactions, which could stabilize packing configurations and connections with other protein molecules, like those in the process of replication complex (**Supplementary Figure S2A**). Q63R (CM.8.1.3), originating in North America and with a frequency of 0.11% in the US. The amino acid Glutamine (Q), which is polar and neutral, is substituted with Arginine (R), which is basic and strongly charged. This change presents a stronger charge that is positive, which could result in improved electrostatic connections to negatively charged compounds, such as RNA or other proteins in the viral replication complex (**Supplementary Figure S2A**). Consequently, the electrical charge and interaction potential of the protein is significantly altered. In the United States, S25L (BQ.1.1) from North America has a frequency of 0.10%. The substitution of a hydrophobic residue (Leucine, L) for a polar side chain (Serine, S) result in a change in the local environment that may improve its interaction with hydrophobic regions of other proteins or membranes by eliminating hydrogen-bonding capacity utilization and incorporating a hydrophobic location (**Supplementary Figure S2A**). The mutation of Leucine to Phenylalanine in position 71 with ΔΔG 1.284 kcal/mol, mutation of Glutamine to Arginine in position 63 with ΔΔG 1.081 kcal/mol and mutation of Serine to Leucine in position 25 with ΔΔG 1.163 kcal/mol cause stabilization of the NSP7 structure (**Supplementary Figure S3**, **Supplementary Table S4**). We identified four mutations in NSP7 that result in the conversion of Leucine to Phenylalanine (L->F). Both Leucine and Phenylalanine are hydrophobic, but the aromatic group of Phenylalanine includes rigidity and can collaborate with other hydrophobic components to alter how the protein folds and connects with them. Leucine to Methionine (L->M) conversion mutations are the most susceptible amino acid conversions in many nations and groups. Since Methionine has sulfur in its side chain, the conversion of Leucine to Methionine (L->M) also adds a bigger hydrophobic residue, which could affect the protein's flexibility and packing. These modifications most likely aid in the stability or modification of protein-protein interactions required for NSP7's function in the replication of the viral complicated (**Supplementary Table S5**).

NSP8 Mutation Track

The NSP8 enzyme has demonstrated the capability to initiate replication de novo and has been suggested to function as a primase, and has been established to exhibit colocalization with RdRp in order to facilitate the replication of the SARS-CoV genome. 198 mutations were discovered in 16 countries which account for 169 mutations for North America, 21 mutations for Europe, 5 mutations for Asia, 2 mutations for South America and 1 mutation for Africa (**Figure 7**). The most frequent mutation in NSP8 was found in the US, N118S (19A), at a frequency of 1.21%. The mutation presents a hydroxyl group at this position, changing the local bonding hydrogen capacity and facilitating more flexible conversations, but it also retains polarity (**Supplementary Figure S2A**). Switzerland, T145I (BQ.1.1.1) at a frequency of 0.57% that the substitution of a heavy hydrophilic side chains for a hydroxyl group results in the mutation of a polar amino acid, Threonine (T), into the hydrophobic, non-polar amino acid, Isoleucine (I). This modification is anticipated to affect local retractable through decreasing polarity and increasing hydrophobic bonds, which could stabilize hydrophobic cores inside the protein (**Supplementary Figure S2A**). England, Q24R (19A) with a frequency of 0.49% that the charge at this site is significantly altered by the substitution of Arginine (R), a positively charged, basic amino acid, for Glutamine (Q), a polar, uncharged amino acid. This mutation presents powerful electrostatic forces and potential bonds of hydrogen, which might improve stability in regions where opposing charges or groups of phosphate are visible (**Supplementary Figure S2A**). The mutation of Asparagine to Serine in position 118 with $\Delta\Delta G$ 1.428 kcal/mol, mutation of Threonine to Isoleucine in position 145 with $\Delta\Delta G$ 1.393 kcal/mol and mutation of Glutamine to Arginine in position 24 with $\Delta\Delta G$ 1.988 kcal/mol cause stabilization of the NSP8 structure (**Supplementary Figure S3**, **Supplementary Table S4**). We detected seven mutations in conversion of Threonine to Isoleucine (T->I), two mutations in conversion of Isoleucine to Threonine (I->T). These mutations include significant shifts among polar and non-polar residues, which are probable to have an influence on hydrophobic packing or hydrogen bonds in the structure of proteins. Lysine to Arginine (K->R) and Arginine to Lysine (R->K) are more moderate changes because both residues are basic, but Arginine has a more complicated side chain that can form more powerful electrostatic bonds. Four mutations in the conversion of Lysine to Arginine (K->R) and two mutations in the conversion of Arginine to Lysine (R->K) are particularly susceptible amino acids that can perform translation in different positions and nationalities for NSP8. These modifications may improve how the protein interacts with RNA or other viral proteins, particularly in charged environments (**Supplementary Table S5**).

NSP9 Mutation Track

NSP9 is a protein that binds to single-stranded RNA, exhibiting a binding fold for oligosaccharides and oligonucleotides. 113 mutations were detected in 12 countries such as 91 mutations for North America, 11 mutations for Europe, 9 mutations for Asia and 2 mutations for Oceania (**Figure 7**). The mutation of Threonine (T), a polar amino acid, to Isoleucine (I), a hydrophobic, non-polar amino acid, eradicates the hydroxyl group and presents a more substantial, hydrophobic side chain for NSP9 mutations, including T35I (BJ.1), which occurs with a frequency of 0.80%. This change from a polar to a hydrophobic residue changes the local environment, which could interfere with connections to RNA or other polar compounds (**Supplementary Figure S2A**). I65V (BA.5.2) from North America that has a frequency of 0.34% in the US. This moderate shift is unlikely to have an important effect on how proteins work but might impact wrapping conversations in the protein's hydrophilic core. Slightly reducing steric restriction in the structure of proteins is the effect of substituting the hydrophobic amino acid Valine (V) for the larger and less side-chained Isoleucine (I) (**Supplementary Figure S2A**). The substitution of Lysine (K), another positively charged residue with a shorter side chain, for Arginine (R), a big, positively charged amino acid, is a conservation alteration. R39K (BN.1.3) has been detected with an incidence of 0.34% in India from Asia. Despite the fact that both residues are basic, Arginine's guanidinium group allows it to make longer bonds of hydrogen, whereas Lysine's electrostatic interactions are easier (**Supplementary Figure S2A**). The mutation of Threonine to Isoleucine in position 35 with ΔΔG -0.257 kcal/mol, mutation of Isoleucine to Valine in position 65 with ΔΔG -0.401 kcal/mol and mutation of Arginine to Lysine in position 39 with ΔΔG -0.017 kcal/mol cause destabilization of the NSP9 structure (**Supplementary Figure S3, Supplementary Table S4**). The process of converting Threonine to Isoleucine (T->I) in NSP9 involves seven mutations that result in a major shift in structure from a polar, hydrogen-bonding residue to a hydrophobic one. This change can decrease the protein's interactions with molecules of water or RNA. The most responsive amino acid conversion that results from three mutations in the conversion of Glycine to Lysine (G->K) in different places and regions. The transition from Glycine to Lysine (G->K) results in the presence of a positively charged residue despite the lack of one previously, which modifies local electrostatic interactions and may improve adhesion to negatively charged molecules such as RNA (**Supplementary Table S5**).

NSP10 Mutation Track

NSP10 induces the activation of nsp16, thereby facilitating the execution of S-adenosyl-L-methionine (SAM)-dependent methyltransferase (MTase) activity. 139 mutations were discovered in 40 countries including 57 mutations for North America, 47 mutations for Europe, 21 mutations for Asia, 7 mutations for Africa, 5 mutations for South America and 2 mutations for Oceania (**Figure 7**). T102I, which has a frequency of 0.16%, and T12I, which has a frequency of 0.12%, are Asian In both mutations, the polar, uncharged amino acid Threonine (T) is replaced with the hydrophobic, non-polar amino acid Isoleucine (I). The protein's ability to form hydrogen bonds is decreased at positions 102 and 12, as the protein shifts from a polar to a hydrophobic environment. This might affect the protein's local interactions with water or polar side chains (**Supplementary Figure S2B**). One of the top three stable variants for the NSP10 protein that has been reported is A104V, which occurs at a frequency of 0.10% in Ecuador, South America. The side-chain size is almost increased by the mutation from the small non-polar amino acid Alanine (A) to the larger, non-polar residue Valine (V) (**Supplementary Figure S2B**). Valine's longer side chain may encourage closer packing within the protein's hydrophilic core, enhancing structural stability regardless of both residues are hydrophobic (**Supplementary Figure S2B**). The mutation of Threonine to Isoleucine in two positions 102 and 12 with ΔΔG 0.389 kcal/mol and ΔΔG 0.564 kcal/mol respectively, and mutation of Alanine to Valine in position 104 with ΔΔG 0.494 kcal/mol cause the stabilization of the NSP10 structure (**Supplementary Figure S3**, **Supplementary Table S4**). Six mutations were found in the conversion of Glycine to Serine (G->S). This conversion creates a polar side chain where none previously was present, opening up the possibility to new hydrogen-bonding interactions that could change the local structure and performance. One mutation in the Serine to Glycine (S->G) conversion does the reverse by eliminating the group of hydroxyls which may cause instability in locations where hydrogen bonding was crucial. Four mutations in the conversion of Cysteine to Serine (C->S) eliminate the side chain carrying sulfur, which may have an impact on the assembly of disulfide bonds or oxidation function. Three changes in the Acid Glutamic to Acid Aspartic (E->D) conversion that the conservative transition from Glutamic Acid to Aspartic Acid (E->D) may slightly modify the spatial connections since Aspartic Acid has a shorter side chain than Glutamic Acid. One mutation that could alter local structure and flexibility in NSP10 is the conversion of Acid Aspartic to Acid Glutamic (D->E), which is the most susceptible amino acid conversion in many locations and countries (**Supplementary Table S5**).

NSP11 Mutation Track

We found 13 mutations in 2 countries which account for 12 mutations for North America and 1 mutation for Europe (**Figure 7**). In the conversion of S6L, the largest mutations in NSP11 were found in Greece, Europe, with a frequency of 0.15% that A substantial alteration in polarity can be brought about by a substitution of Leucine (L), a non-polar, hydrophobic amino acid, from Serine (S), a small polar amino acid (**Supplementary Figure S2B**). Leucine replaces serine, removing the hydroxyl group that forms hydrogen bonds and adding a large, hydrophobic side chain in its place. This alteration most likely increases hydrophobic interactions within the protein core while decreasing the protein's interactions with water or other polar residues. Mutation N9S, which is 0.04% common in the US and originates in North America, the side chain's potential for hydrogen bonding is altered when Serine (S), a smaller polar amino acid, replaces Asparagine (N), a polar amino acid that can form hydrogen bonds. Although hydrogen bonds can form between both residues, Asparagine's amide group permits longer-lasting connections than Serine's hydroxyl group. This mutation may cause local networks of hydrogen bonds to separate (**Supplementary Figure S2B**). S1A mutation with a frequency of 0.03% in North America's United States that the hydroxyl group, which is essential for hydrogen bonding, is eliminated when Serine (S) is mutated into the non-polar amino acid Alanine (A). The protein's polarity is decreased as a result, which may influence how it interacts with adjacent polar residues or water molecules. However, in some hydrophobic locations, Alanine's small size may allow for greater packing (**Supplementary Figure S2B**). The mutation of Serine to Leucine in position 6 with ΔΔG 0.233 kcal/mol cause stabilization, and the mutation of Asparagine to Serine in position 9 with ΔΔG -0.268 kcal/mol cause destabilization of the NSP11 structure (**Supplementary Figure S3**, **Supplementary Table S4**). Although Valine and Alanine are both non-polar and hydrophobic, but Alanine has a larger side chain, we found two mutations in the conversion of Alanine to Valine (A->V). These amino acids are particularly sensitive to conversion in distinct sites and positions for NSP11. The structure of the protein folds or packs could be altered by this size increase, which could have an impact on how it interacts with other hydrophobic locations. These modifications might affect the local rigidity and packing density in the hydrophobic core but are unlikely to have a significant effect on the structure overall (**Supplementary Table S5**).

NSP12 Mutation Track

The viral replication complex is constituted by NSP12, which operates in conjunction with nsp7, nsp8, and other indispensable constituents of the RNA synthesis machinery. 932 mutations were discovered in 34 countries which account for 801 mutations for North America, 76 mutations for Europe, 25 mutations for South America, 21 mutations for Asia, 8 mutations for Africa and 1 mutation for Oceania (**Figure 7**). The mutation from Proline (P), a stiff, non-polar amino acid, to Leucine (L), a more flexible hydrophobic amino acid, increases the flexibility of the structure of proteins. This is one of the greatest mutations of NSP12 that has been found, with a frequency of 99.4% in Switzerland. Leucine permits a more typical alpha-helix or beta-sheet shape, but Proline's cyclic nature causes kinks in protein structures (**Supplementary Figure S2B**). In the Gambia, G671S mutation with a frequency of 33.9% from Africa. A hydroxyl group appears where none previously existed when the smallest amino acid, Glycine (G), is mutated into the little polar amino acid, Serine (S). While Serine adds the potential for hydrogen bonds, which could limit local flexibility, Glycine offers flexibility because it does not contain a side chain (**Supplementary Figure S2B**). Y273H from Europe, which has a frequency of 3.05% in Norway. There is a major functional shift when Histidine (H), a basic, polar amino acid, replaces Tyrosine (Y), a polar, aromatic amino acid. The hydroxyl group of Tyrosine can engage in the bonding of hydrogen, and depending on pH, Histidine might produce a positively charged side chain that may change electrostatic connections (**Supplementary Figure S2B**). The mutation of Proline to Leucine in position 323 with ΔΔG 0.583 kcal/mol and mutation of Tyrosine to Histidine in position 273 with ΔΔG 0.001 kcal/mol cause the stabilization of the protein structure. The mutation of Glycine to Serine in position 671 with ΔΔG -0.287 kcal/mol cause destabilization of the NSP12 structure (**Supplementary Figure S3**, **Supplementary Table S4**). Ten mutations were found to be in the conversion of Phenylalanine to Leucine (F->L) and eight mutations were found to be in the conversion of Leucine to Phenylalanine (L->F). Both of these exchanges are hydrophobic, which but the aromatic chain of Phenylalanine adds the majority and stiffness, which may have an impact on protein being packed. Eight mutations happened though converting Alanine to Serine (A->S), and six occur when converting Serine to Alanine (S->A). These mutations involve small non-polar to polar shifts, which may include or remove hydrogen-bonding potential and affect regional interactions and folding. The conversion of amino acids in different locations and nations as extremely sensitive in NSP12. Five mutations in the conversion of Glycine to Serine (G->S) and four mutations in the conversion of Serine to Glycine (S->G). Mutations have an impact on flexibility; Serine adds strength because of its hydroxyl group, but Glycine is quite flexible because it lacks a side chain (**Supplementary Table S5**).

NSP13 Mutation Track

The NSP13 protein is capable of unwinding double-stranded RNA or DNA in a manner that proceeds with a 5' to 3' polarity. This process is made possible through the utilization of energy that is derived from nucleotide hydrolysis. 601 mutations were discovered in 24 countries including 504 mutations for North America, 61 mutations for Europe, 28 mutations for Asia, 5 mutations for South America, 2 mutations for Africa and 1 mutation for Oceania (**Figure 7**). NSP13 mutations, such as R392C (XBB.1.5), which occur with a frequency of 35.76% in the Netherlands. A substantial chemical change is brought about by the transformation of the large, positively charged amino acid Arginine (R) to the small, polar amino acid Cysteine (C), which has a thiol group that contains sulfur. Cysteine, a redox-active amino acid, takes over Arginine's capacity for hydrogen bonding and strong electrostatic interactions (**Supplementary Figure S2B**). Disulfide linkages that Cysteine can generate can change whether proteins fold. P77L (B.1) from Europe, with a frequency of 29.70% in the Netherlands, that Flexibility is added by replacing the stiff, non-polar amino acid Proline (P) with the more flexible hydrophobic amino acid Leucine (L). Because of its cyclic nature, Proline places structural restrictions on proteins, but Leucine offers more conformation independence, which may improve folding of proteins and assembly (**Supplementary Figure S2B**). M233I (BQ.1.1) from Asia, which has a frequency of 3.80% in India. The change from the non-polar amino acid Methionine (M) to the identically non-polar but larger Isoleucine (I) includes a minor but important structural modification. The sulfur in Methionine may be involved in delicate interactions, whereas the bigger hydrophobic side chain of Isoleucine might improve wrapping in hydrophobic regions, facilitating the structural stability of the protein (**Supplementary Figure S2B**). The mutation of Arginine to Cysteine in position 392 with $\Delta\Delta G$ 1.127 kcal/mol, mutation of Proline to Leucine in position 77 with $\Delta\Delta G$ 1.101 kcal/mol and mutation of Methionine to Isoleucine in position 233 with $\Delta\Delta G$ 1.688 kcal/mol cause the stabilization of NSP13 structure (**Supplementary Figure S3**, **Supplementary Table S4**). We identified four mutations in the conversion of Isoleucine to Threonine (I->T) and eleven mutations in the conversion of Threonine to Isoleucine (T->I). differences in the percentage of Threonine to Isoleucine (T->I) and Isoleucine to Threonine (I->T) require large movements between polar and non-polar residues, which may have an impact on chemical packing, local folding, and flexibility. Glutamic Acid to Aspartic Acid (E->D) and Aspartic Acid to Glutamic Acid (D->E) are conservative changes that both involve negatively charged acidic residues; however, Glutamic Acid has a more side chain, which may have an impact on the arrangement of space and connection with other residues or molecules. Five mutations in the conversion of Acid Glutamic to Acid Aspartic (E->D) and two mutations in the conversion of Acid Aspartic to Acid Glutamic (D->E) in distinct positions and locations displaying a significant susceptibility to amino acid conversion for NSP13 (**Supplementary Table S5**).

NSP14 Mutation Track

The N-terminal exoribonuclease domain of NSP14 plays a crucial role in proofreading, thereby preventing the occurrence of lethal mutagenesis. Conversely, the C-terminal domain of NSP14 functions as a (guanine-N7) methyltransferase (N7-MTase), which is responsible for mRNA capping. 527 mutations were detected in 29 countries which account for 463 mutations for North America, 33 mutations for Europe, 23 mutations for Asia, 4 mutations for South America, 2 mutations for Africa and 2 mutations for Oceania (**Figure 7**). The most common mutation in NSP14 was found in conversion I42V (XBB.1.5), which occurred with frequency 55.56% in the United States. This mutation is a cautious one, changing from the hydrophobic, heavy amino acid Isoleucine (I) to the slightly less hydrophobic Valine (V). Both residues are non-polar and involved in hydrophobic interactions, but Valine's smaller side chain could make way for more precise packing in the central hydrophobic region (**Supplementary Figure S2B**). A394V (AY.103) in Guangdong, China, that has a frequency of 23.51%. The inclusion of the majority to the protein core results from the substitution of the larger a hydrophobic residue Valine (V) for the smaller, non-polar amino acid Alanine (A). Although both residues are hydrophobic, Valine's larger side chain may enhance hydrophobic bonds in this area, thus helping to stabilize the structure of the protein (**Supplementary Figure S2B**). N129D (XBB) from North America, which has a frequency of 0.91% in the US. This mutation results in a significant charge change at this location: Aspartic Acid (D), a negatively charged amino acid, replaces Asparagine (N), a polar, uncharged amino acid. Aspartic Acid may interfere with protein interactions due to its negative charge, whereas Asparagine may engage in bonds of hydrogen. This mutation probably has an impact on contacts with other residues or local structure (**Supplementary Figure S2B**). The mutation of Isoleucine to Valine in position 42 with $\Delta\Delta G$ 1.243 kcal/mol, mutation of Alanine to Valine in position 394 with $\Delta\Delta G$ 0.991 kcal/mol cause stabilization of the protein structure. The mutation of Asparagine to Acid Aspartic in position 129 with $\Delta\Delta G$ -0.056 kcal/mol cause destabilization of the NSP14 structure (**Supplementary Figure S3**, **Supplementary Table S4**). We found that there were eight mutations in NSP14 that affected the conversion of Alanine to Valine (A->V) and five mutations that affected the conversion of Valine to Alanine (V->A). Both of these mutations involved cautious a hydrophobic provides that affected organizing and hydrophilic. Acid Aspartic to Tyrosine (D->Y) conversion has seven mutations, while the Tyrosine to Acid Aspartic (Y->D) conversion contains three mutations. Both the Aspartic Acid to Tyrosine (D->Y) and Tyrosine to Aspartic Acid (Y->D) reactions involve significant changes because the vulnerability of amino acid converting varies across various locations and countries. Tyrosine is polar and aromatic and can form bonds of hydrogen, whereas Aspartic Acid begins a negatively charged side chain. These alterations are probably going to impact stability, local structure shape, and electrostatic connections (**Supplementary Table S5**).

NSP15 Mutation Track

The enzyme NSP15 exhibits a preference for cleaving uridines at the 3' position, a process that is reliant on the presence of manganese. This mechanism is widely believed to play a crucial role in the evasion of antiviral defense by the virus. 346 mutations were discovered in 25 countries such as 294 mutations for North America, 30 mutations for Europe, 11 mutations for Asia, 8 mutations for South America, 2 mutations for Africa and 1 mutation for Oceania (**Figure 7**). T112I (XBB.1.5), one of the mutations, has a frequency of 34.60% in England. The hydroxyl group that is involved in the formation of hydrogen bonds is removed and replaced with a large hydrophobic side chain when Threonine (T), a polar amino acid, gets mutated into Isoleucine (I), a hydrophobic, non-polar amino acid. Destabilization results from this alteration, which probably decreases hydrogen bonds and increases hydrophobic interactions in the protein (**Supplementary Figure S2B**). The replacement of Valine (V), a larger hydrophobic residue, for Alanine (A), a small, non-polar amino acid, begins a larger side chain lacking significantly modifying the chemical properties. A80V (BQ.1) has a frequency of 0.31% in the United States. This sensible mutation has the potential to enhance organizing in hydrophilic areas of the protein, facilitating stabilizing (**Supplementary Figure S2B**). K259R (BN.1), with a frequency of 0.19% in England, is one of the top three stable mutations for NSP15 that have been recorded in various locations and nations. A shorter side chain is swapped out for Arginine's larger, more complicated guanidinium group during the mutation from Lysine (K) to Arginine (R), both positively charged amino acids. Compared to Lysine, Arginine can establish stronger hydrogen bonds and electrostatic connections, which may have a little influence on the regional conformation (**Supplementary Figure S2B**). The mutation of Threonine to Isoleucine in position 112 with ΔΔG -0.282 kcal/mol and mutation of Lysine to Arginine with ΔΔG -0.071 kcal/mol cause destabilization of the protein structure. The mutation of Alanine to Valine in position 80 with ΔΔG 3.275 kcal/mol cause stabilization of the NSP15 structure (**Supplementary Figure S3, Supplementary Table S4**). Eight mutations were found to take place in the conversion of Valine to Leucine (V->L), while four mutations were found to exist in the conversion of Leucine to Valine (L->V). These mutations represent cautious modifications between hydrophobic residues, with very minor variations in side-chain length. Although major structural instabilities are anticipated to result from these modifications, hydrophilic packing may be improved. Two mutations in the conversion of Lysine to Phenylalanine (K->F) and three mutations in the conversion of Phenylalanine to Lysine (F->K). This conversions in NSP15 involves more dramatic changes among a big, non-polar aromatic residue and a positively charged amino acid, with different locations and countries exhibiting a high vulnerability to these changes. These modifications would profoundly modify electrostatic bonds, which could have an impact on the stability of proteins or affinity for binding (**Supplementary Table S5**).

NSP16 Mutation Track

The recruitment of N7-methylated capped RNA and SAM by NSP16 serves to facilitate the assembly of the enzymatically active nsp10/nsp16 complex. This process is crucial for the successful execution of the complex's enzymatic functions. 298 mutations were detected in 59 countries which account for 122 mutations for Europe, 102 mutations for North America, 49 mutations for Asia, 14 mutations for South America, 9 mutations for Africa and 2 mutations for Oceania (**Figure 7**). The majority of the mutations, K160R (BQ.1.1.5), with a frequency of 1.10% for NSP16, were found in Wuhan, China. This mutation is a cautious modification that results in Arginine (R), another basic amino acid with a longer and more complicated side chain, replacing Lysine (K), an amino acid that is basic and positively charged (**Supplementary Figure S2B**). In comparison with Lysine, Arginine's guanidinium group, which can create stronger electrostatic connections, gives it a greater potential for bonding through hydrogen. A substantial modification in size and chemical characteristics is shown by the replacement of Cysteine (C), a polar amino acid with a thiol group, for positively charged Arginine (R). This alteration eliminates a positively charged residue and replaces it with a smaller side chain that is capable of establishing disulfide bonds. The R216C (EA.1) mutation for NSP16 was discovered in the United States from North America at a frequency of 0.95% (**Supplementary Figure S2B**). The Q238H (BQ.1) mutation with frequency 0.69% for NSP16 was observed in Japan, Asia. The amino acid Glutamine (Q), which is polar and free of charge, can mutate into Histidine (H), which is polar and can be positively charged according to pH. This mutation changes the potential for electrostatic attraction and hydrogen bonding between the two amino acids. Histidine's imidazole group can participate in proton substitution and collaborate with encompassing molecules, but it may also alter the regional structure through adding an alternative charge potential (**Supplementary Figure S2B**). The mutation of Lysine to Arginine in position 160 with $\Delta\Delta G$ 0.305 kcal/mol cause stabilization of the protein structure. The mutations such as Arginine to Cysteine in position 216 with $\Delta\Delta G$ -0.373 kcal/mol and Glutamine to Histidine in position 238 with $\Delta\Delta G$ -0.398 kcal/mol cause destabilization of the NSP16 structure (**Supplementary Figure S3**, **Supplementary Table S4**). Five mutations in the conversion of Glycine to Serine (G->S) and one mutation in the conversion of Serine to Glycine (S->G) were found for NSP16. The alteration of Glycine to Serine (G->S) provides a polar side chain where none previously existed, which may have an impact on hydrogen-bonding contacts and flexibility within the protein. Five mutations in the Histidine to Tyrosine (H->Y) conversion and four mutations in the Tyrosine to Histidine (Y->H) conversion demonstrating the conversion of amino acids' vulnerability. It is clear that changes between polar, aromatic residues in Histidine to Tyrosine (H->Y) and Tyrosine to Histidine (Y->H) can impact interactions between proteins in a variety of locations and nations. While the imidazole group of Histidine can be protonated, Tyrosine's hydroxyl group can form hydrogen bonds, which may change the stability and electrostatic interactions in these locations (**Supplementary Table S5**).

ORF6 Mutation Track

The suppression of both interferon (IFN) induction and signaling pathways is a function attributed to ORF6. 61 mutations were detected in 20 countries including 26 mutations for Europe, 20 mutations for North America, 9 mutations for Asia, 5 mutations for South America and 1 mutation for Oceania (**Figure 7**). The top three Orf6 mutations were found in England, including D61L with a frequency of 19.92%. This mutation substitutes a hydrophobic side chain for a charged one, going from Aspartic Acid (D), a negatively charged polar amino acid, to Leucine (L), a non-polar amino acid. Hydrophobic interactions are anticipated to replace electrostatic interactions and hydrogen bonding as a result of this modification. In hydrophobic settings, the substitution is anticipated to encourage local stability, especially in areas connected to membranes. The substitution of Asparagine (N), a polar, uncharged amino acid, for Lysine (K), a positively charged amino acid, results in a loss of charge at position 48 (**Figure 8**). This mutation of K48N (BJ.1), with a frequency of 0.31%, was observed in England. Asparagine is mainly involved in hydrogen bonding, whereas Lysine's positive charge is essential for electrostatic interactions. The protein's capacity to interact with nucleic acids or other proteins may be affected by this mutation, especially in areas where electrostatic binding is necessary (**Figure 8**). The P57L mutation, which is present in England at a frequency of 0.17% in Europe, at position 57, conformational flexibility is created by a mutation from the fixed, cyclical amino acid Proline (P) to the flexible, hydrophobic residue Leucine (L). Proline usually causes kinks or shifts in the protein backbone, so Leucine's replacement is probable to release these limitations and stabilize additional structures like alpha-helices or beta-sheets (**Figure 8**). Six mutations in the conversion of Isoleucine to Valine (I->V) and one mutation in the conversion of Valine to Isoleucine (V->I) were found in ORF6. These mutations are cautious replacements between hydrophobic residues, with only slight variations in the overall size and flexibility of the side chains. Although major structural instabilities are unlikely to result from these modifications, local hydrophobic packing may be improved. Isoleucine to Threonine (I->T) conversion has four mutations. Isoleucine to threonine (I->T) begins a polar side chain where there was none before, which might disrupt hydrophobic bonds and affect local hydrogen bonding, potentially altering stability in certain regions due to the varying susceptibility of amino acid conversion in different locations and countries (**Supplementary Table S5**).

ORF9b Mutation Track

The protein ORF9b demonstrate interaction with certain nonstructural proteins and subsequently integrates into fully developed virions. 97 mutations were found in 30 countries which account for 41 mutations for Europe, 34 mutations for North America, 11 mutations for Asia, 6 mutations for Africa, 4 mutations for South America and 1 mutation for Oceania (**Figure 7**). The conversion of T60A, which has a frequency of 61.06% in France, Europe, is the most common mutation in ORF9b. The side-chain size is lowered and the hydrogen-bonding potential is eliminated when Threonine (T), a polar amino acid containing a hydroxyl group, is changed to Alanine (A), a smaller non-polar amino acid. This alteration probably contributes to the observed destabilization of the ORF9b structure by weakening polar contacts, which loosens the local protein structure (**Figure 8**). S6C from North America that is 4.37% frequent in Canada. Disulfide bond formation is possible when Serine (S), a polar amino acid, is substituted with Cysteine (C), an amino acid that contains sulfur. This modification has the ability to drastically modify protein structure through the creation of disulfide bonds (**Figure 8**). The mutation from the polar amino acid Glutamine (Q) to the negatively charged amino acid Glutamic Acid (E) provides a charge at position 77 (Q77E, frequency 1.85% in Colombia, South America). This mutation changes local electrostatic interactions, most probably destabilize the protein (**Figure 8**). The mutation of Threonine to Alanine in position 60 with $\Delta\Delta G$ -0.171 kcal/mol and mutation of Glutamine to Acid Glutamic in position 77 with $\Delta\Delta G$ -0.169 kcal/mol cause destabilization of the ORF9b protein structure (**Supplementary Figure S3**, **Supplementary Table S4**). We found six mutations in ORF9b that resulted in the conversion of Valine to Leucine (V->L). This substitution is conservative because it involves two hydrophobic residues and very slightly alters the majority and flexibility of the side chains, perhaps preserving hydrophobic core interactions. Five mutations in the conversion of Proline to Leucine (P->L) increase the flexibility of the local structure by removing the inflexible proline backbone limitation. Four mutations in the Leucine-to-Proline (L->P) conversion cause stiffness, which may have an effect on secondary structures and folding of proteins. Three mutations in the conversion of Leucine to Serine (L->S) result in the begins of a polar side chain, which disrupts hydrophobic areas and may cause structural instability. One mutation that affects the most vulnerable amino acid, Serine to Leucine (S->L), promotes hydrophobic packing but decreases flexibility in a variety of places and geographical areas (**Supplementary Table S5**).

ORF9c Mutation Track

73 mutations were detected in 27 countries including 38 mutations for Europe, 19 mutations for North America, 6 mutations for Asia, 6 mutations for Africa, 3 mutations for South America and 1 mutation for Oceania (**Figure 7**). With a frequency of 64.45%, the most common mutation G50N for ORF9c was found in England, Europe. A long side chain with hydrogen-bonding capability is introduced by the mutation of the smallest amino acid, Glycine (G), to the bigger polar amino acid, Asparagine (N). This modification probably enhances local rigidity of structure through the begins of hydrogen bonds, which can influence protein dynamics and folding (**Figure 8**). The change from Valine (V), a small hydrophobic amino acid, to Leucine (L), a heavy hydrophobic amino acid, significantly increases the size of the side chain while preserving hydrophobic interactions. This mutation of V49L for ORF9c was observed in England with a frequency of 1.80%. Although the stability and function of proteins are probably not going to be significantly affected by this cautious replacement, in some circumstances it might modestly enhance hydrophobic packing (**Figure 8**). The mutation of L52F for ORF9c was observed in England with a frequency of 1.33%. The substitution of Phenylalanine (F), an aromatic amino acid, for Leucine (L), a hydrophobic amino acid, presents a larger, rigid naturally occurring engagement ring. This modification increases stiffness and might generate stacking connections, altering the hydrophobic core packing and potentially affecting protein function or interactions (**Figure 8**). Six changes in the conversion of Alanine to Valine (A->V) for ORF9c were identified. Valine introduces a little amount more mass than Alanine, which could enhance hydrophobic interactions. Alanine to Valine (A->V) is a conservative substitution between two small, hydrophobic residues. A single mutation in the conversion of Valine to Alanine (V->A) reverses this by minimizing the side chain's size. Five mutations in the conversion of Leucine to Phenylalanine (L->F) result in the begins of an aromatic ring, which increases stiffness and raises the possibility of stacking interactions. The most vulnerable amino acid conversion occurs when three mutations are present in the conversion of Acid Glutamic to Lysine (E->K) and one mutation occurs in the conversion of Lysine to Acid Glutamic (K->E). The former greatly alters electrostatic interactions by introducing a positive charge in place of a negative charge, while the other (K->E) replaces a positive charge with a negative one, which could impact protein structure and connections (**Supplementary Table S5**).
