# Supplementary figures and images for: Spatial Transcriptomics and Single Cell‐RNASeq Reveals Cellular Heterogeneity of SARS‐CoV‐2 in Lung Tissues and Global Mutational Patterns in COVID‐19 Patients
Source: J Med Virol. 2025 Sep 5;97(9):e70586. doi: 10.1002/jmv.70586 (PMC12412077; doi:10.1002/jmv.70586)

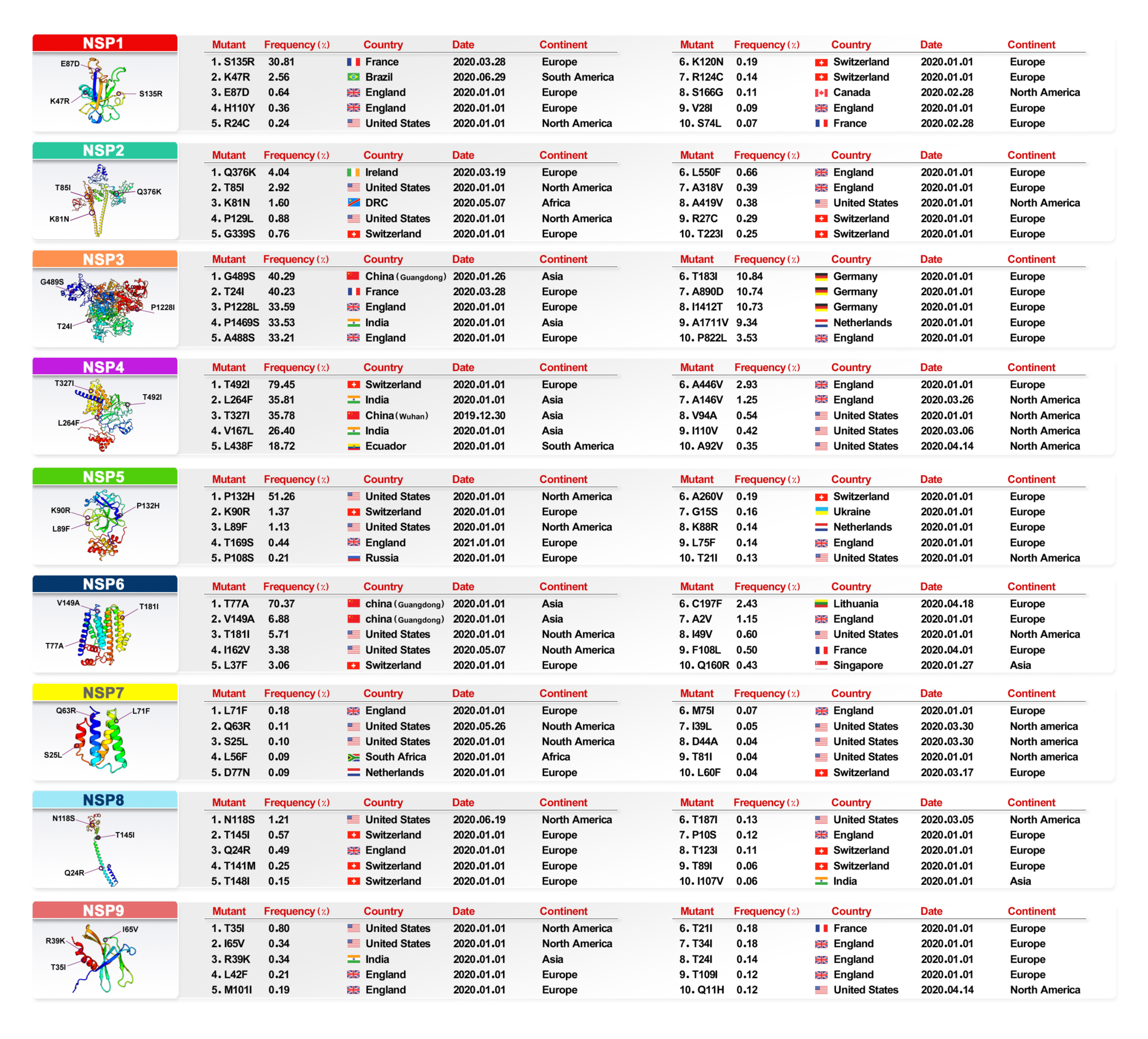

Supplement: Supplementary file 3 — Supplementary Figure S2A: The top 10 mutations of genes (NSP1‐NSP9) such as: mutation position, mutation frequency, country name, mutation date and continent. [file JMV-97-e70586-s008.jpg]

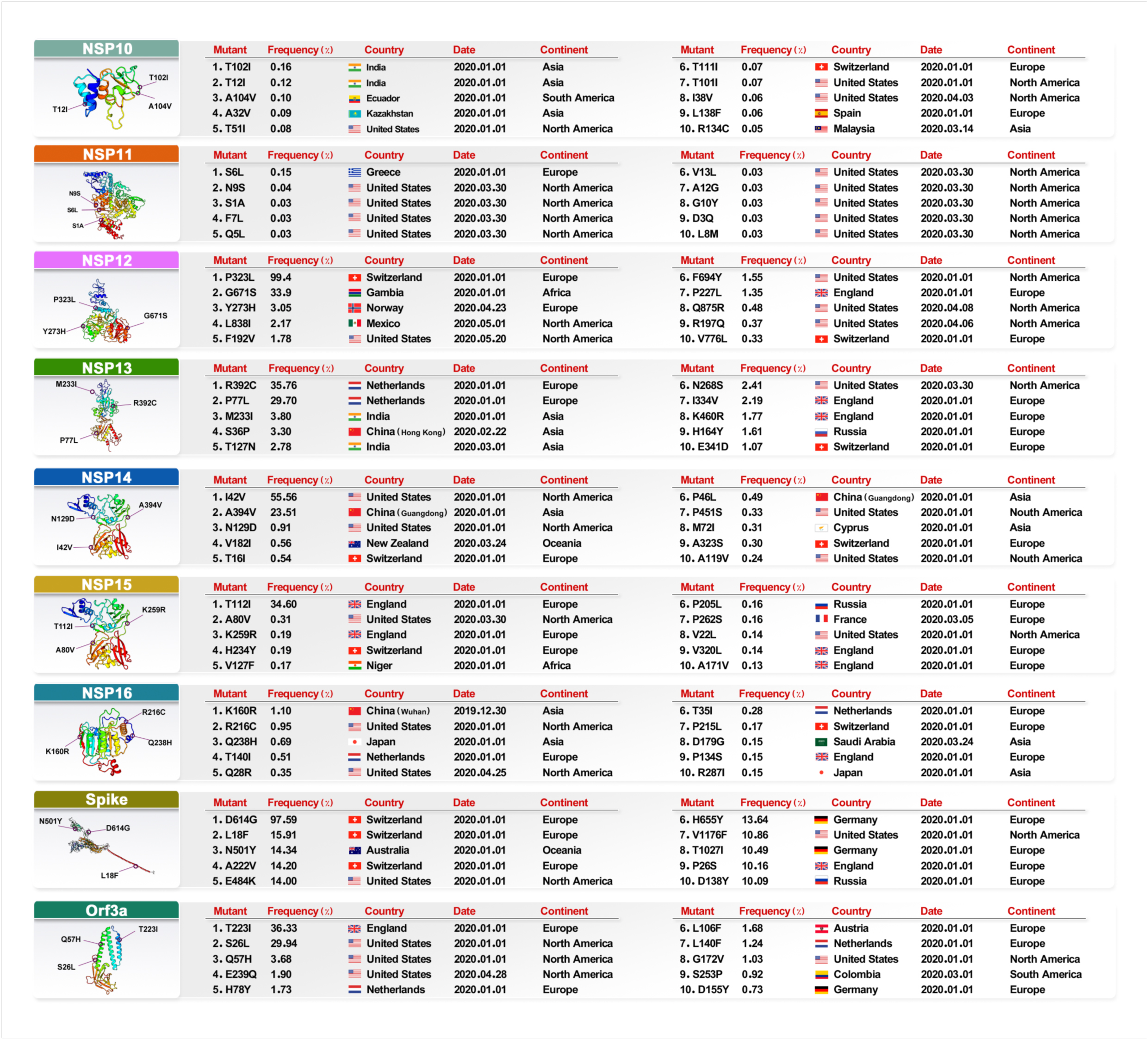

Supplement: Supplementary file 4 — Supplementary Figure S2B: The top 10 mutations of genes (NSP10‐NSP16, Spike and ORF3a) such as: mutation position, mutation frequency, country name, mutation date and continent. [file JMV-97-e70586-s001.jpg]

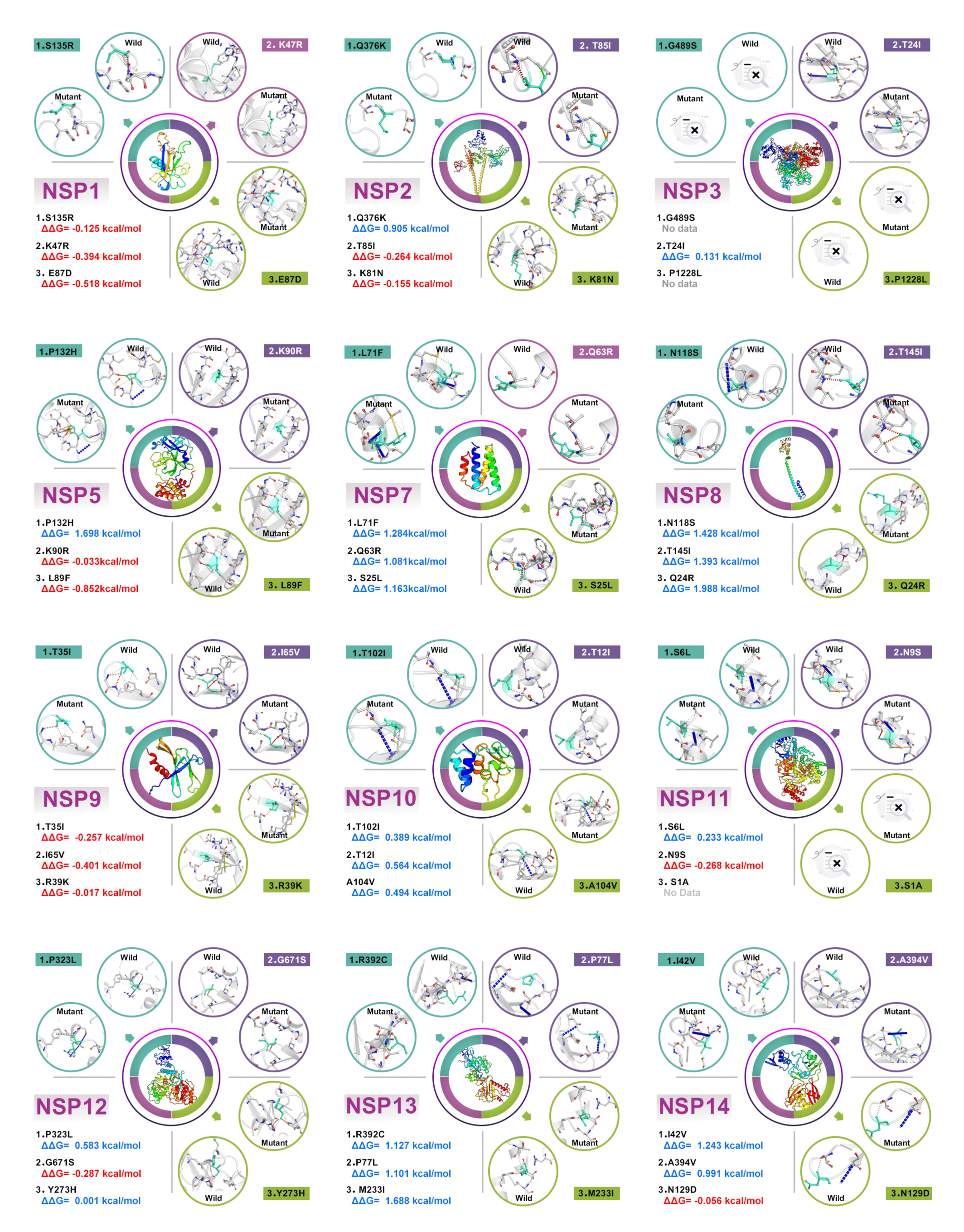

Supplement: Supplementary file 5 — Supplementary Figure S3: Results of the DynaMut server to investigate the effect of mutations on the stability or instability of the SARS‐CoV‐2 proteins including NSP1 to NSP14. V10B13‐401. [file JMV-97-e70586-s006.jpg]
