# Supplementary material for: Spatial Transcriptomics and Single Cell‐RNASeq Reveals Cellular Heterogeneity of SARS‐CoV‐2 in Lung Tissues and Global Mutational Patterns in COVID‐19 Patients
Source: J Med Virol. 2025 Sep 5;97(9):e70586. doi: 10.1002/jmv.70586 (PMC12412077; doi:10.1002/jmv.70586)
Supplement: Supplementary file 6 — Supplementary Fiugre's Caption. [file JMV-97-e70586-s007.pdf]

Supplementary Fiugre's Captions

Supplementary Figure S1. Spatial autocorrelation analysis of viral gene expression in lung tissue samples from COVID-19 patients. Moran's I scatter plots display the degree of spatial clustering for key viral genes across **(A)** sample V10B13-401 and **(B)** sample V10L13-003. Statistically significant spatial patterns ($p < 0.05$) are observed for multiple genes in both samples, with stronger clustering generally seen in sample V10B13-401. Genes with non-significant spatial autocorrelation suggest more diffuse or random distribution within the tissue. These results highlight the heterogeneity of viral gene localization in infected lung tissues.

Supplementary Figure S2A. The top 10 mutations of genes (NSP1-NSP9) such as: mutation position, mutation frequency, country name, mutation date and continent

Supplementary Figure S2B. The top 10 mutations of genes (NSP10-NSP16, Spike and ORF3a) such as: mutation position, mutation frequency, country name, mutation date and continent.

Supplementary Figure S3. Results of the DynaMut server to investigate the effect of mutations on the stability or instability of the SARS-CoV-2 proteins including NSP1 to NSP14.
